# Supplementary material for: Real-time observation of a metal complex-driven reaction intermediate using a porous protein crystal and serial femtosecond crystallography
Source: Nat Commun. 2024 Jun 29;15:5518. doi: 10.1038/s41467-024-49814-9 (PMC11217357; doi:10.1038/s41467-024-49814-9)
Supplement: Supplementary file 1 — Supplementary Information [file 41467_2024_49814_MOESM1_ESM.pdf]

Supplementary Information

Real-time observation of a metal complex-driven reaction intermediate using a porous protein crystal and serial femtosecond crystallography

Basudev Maity,[1*] Mitsuo Shoji,[2*] Fangjia Luo,[3] Takanori Nakane,[4] Satoshi Abe,[1] Shigeki Owada[3,5] Jungmin Kang,[5] Kensuke Tono,[3,5] Rie Tanaka,[5,6] Thuc Toan Pham,[1] Mariko Kojima,[1] Yuki Hishikawa,[1] Junko Tanaka,[1] Jiaxin Tian,[1] Misaki Nagama,[1] Taiga Suzuki,[1] Hiroki Noya,[1] Yuto Nakasuji,[1] Asuka Asanuma,[1] Xinchen Yao,[1] So Iwata,[5,6] Yasuteru Shigeta,[2] Eriko Nango,[5,7*] Takafumi Ueno.[1,8*]

[1] *School of Life Science and Technology, Tokyo Institute of Technology, Nagatsuta-cho 4259, Midori-ku, Yokohama, Japan.*

[2] *Center for Computational Sciences, University of Tsukuba, 1-1-1 Tennodai, Tsukuba, Ibaraki 305-8577, Japan.*

[3] *JASRI, 1-1-1, Kouto, Sayo-cho, Sayo-gun, Hyogo 679-5198 Japan*

[4] *Institute of protein research, Osaka University.*

[5] *RIKEN SPring-8 Center, Hyogo 679-5148, Japan*

[6] *Department of Cell Biology, Graduate School of Medicine, Kyoto University.*

[7] *Tohoku University. Institute of Multidisciplinary Research for Advanced Materials, Tohoku University*

[8] *Research Center for Autonomous Systems Materialogy (ASMat) Institute of Innovative Research, Tokyo Institute of Technology, Nagatsuta-cho 4259, Midori-ku, Yokohama, Japan*

Supplementary Methods

Structure refinement

All the structure refinements were done with the Phenix suite.[1] The protein models were located by Phaser[2] using the PDB entry 193L[3] as a starting model. The model building was done in COOT followed by refinement in phenix.refine. The processes were continued until a reasonable model was built. Water molecules were modelled automatically in COOT with a cutoff difference density map at 3.0 σ and weak density waters were modelled manually. Highly coordinated water molecules at Asp35 and Asp101 in some structures were tentatively assigned as Na^+ ions. Sidechains of the residues Gln121, Arg125 and Arg128 in some structures were truncated due to lack of sufficient density.

The presence of $[Mn(CO)_3(wat)_2]^+$ moiety in the structure under darkness was identified by comparing with a metal-free HEWL structure. To confirm this, we performed a similar reaction using a single crystal which gave a similar density map, and the Mn ion was confirmed by anomalous density at 6σ (Supplementary Figure 1). This is consistent with the previous report on HEWL-$Mn(CO)_3$ structure.[4] The assignment of $Mn(CO)_3(wat)_2$ moiety was further validated by omit map (phase bias) and Polder map (bulk solvent contribution). The structures of $Mn(CO)_3(wat)_2$ after light irradiation was modelled based on the combination of $2F_o$-F_c maps (F_o and F_c are the observed and calculated structure factor amplitude) and isomorphous difference maps with dark structures. During the initial stages of refinement, both B-factor (displacement of the atomic position from an average (mean) value(mean-square displacement)) and occupancy were allowed to refine freely. In the later stages of model building, the occupancy values at Mn center were adjusted to keep the B-factor of Mn ion close to the coordinating atom (N^{ε}) of His15. The resulting Mn coordination structures were validated using the check my metal server (https://cmm.minorlab.org/).

Difference maps (F_o-F_o) were calculated by the "Isomorphous difference map" task in the Phenix GUI. A complete darkness data was collected each beam time and used as a phase and amplitude reference. It is to be noted that since the interleaved dark data were collected in between two light data sets (see the setting in Fig-4b), there is a possibility of light contamination in interleaved dark data. Therefore, we always considered the difference maps obtained against the complete darkness data in which no laser was used. To evaluate the light contamination, difference maps against interleaved dark were calculated as shown in Supplementary Figure 6a in SI. In addition, we also performed the experiment with negative delay in which light was irradiated after XFEL pulse (Supplementary Figure 6b,c).

For quantification, difference density maxima were measured in COOT in units of e/Å3 as well as σ (root mean square electron density of the unit cell) in Supplementary Table 6 but were assigned as zero if features were not identifiably as distinct peak.[5] Similarly, the omit map densities were also

measured to estimate the apparent occupancy of the released CO or Mn. The data are plotted and shown in Supplementary Figure 9.

All the structures were validated in Molprobity[6] and deposited in wwPDB server with accession code (8WZF, 8WZG, 8WZR, 8WZT and 8WZV).

Validity of the QM/MM theoretical level

Dependences of the theoretical condition such as basis set, DFT functional and QM region to the peak top of the absorption spectra and the first excited energy are shown in Supplementary Table 7. RMSD values of the Mn center by using larger basis sets are 0.228Å(DZVP), 0.216Å(TZVP), and 0.216 Å(TZVP+) compared to the X-ray structure (Supplementary Figure 13). This result means that the geometrical structures are only improved by 0.008 Å, even for the larger basis set. On the other hand, the excited state property, such as peak-top wave number around 400 nm, is very sensitive to the basis sets (384(entry 2) – 387(entry 1) = –3 nm), DFT functional (389 (entry 6) – 386 (entry 3) = +3 nm) and QM region (394(entry 9)- 389(enety6) = 5 nm). Even though the optimized geometry by using TZVP(B2) does not improve for the peak-top wavelength compared to the structures calculated by using DZVP (394(entry12)-394(entry9) = 0 nm), considering the computational costs, large number of structural optimizations should be performed at the B3LYP/DZVP(B1) theoretical level, while the few UV spectra calculations can be carried out at the larger basis set and larger QM region as the same level of entry 9 in Supplementary Table 7.

Comparison between QM/MM optimized structures and TR-SFX structures

Direct comparisons of the bond distances around the Mn center and root-mean-square deviation (RMSD) among the QM/MM optimized structures and the SFX results are shown in Supplementary Table 5 and 6. The average Mn-C coordination length of the QM/MM optimized structure **1** is 1.81 Å, slightly shorter compared to the corresponding one of 1.94 Å in SFX result (PDBID:8WZF). The average Mn-O coordination length in QM/MM structure **1** is 2.12Å corresponding to the SFX (PDBID:8WZF) result of 2.24 Å. In both cases, the difference between QM/MM and SFX results is about -0.12 Å, and this trend is very similar in other states. From the RMSD values around the Mn center (Supplementary Table 6), the closest SFX structures from QM/MM structures (**1**-**3**) are commonly complete dark SFX model (**8WZF**), whereas the closest QM/MM structures from SFX structures become **1**, **2** and **3** for SFX structures of (**8WZF**) complete dark, (**8WZG**) 10ns 20μJ (**8WZV**) 1μs 40μJ, respectively. Larger RMSD values of around 0.2 Å come from the larger disorder at the equatorial water ligands, not CO ligands. This implies the flexibility of the water coordination. The RMSD results indicate that structural similarity is highest in the initial non-photoexcited state, and the structural similarity becomes relatively worse as the reaction proceeds after the

photoexcitation. It should be emphasized that the closest QM/MM structure in actual species corresponds best to the SFX structure. Based on these detailed structural comparisons, SFX structures reflect the photochemical reaction.

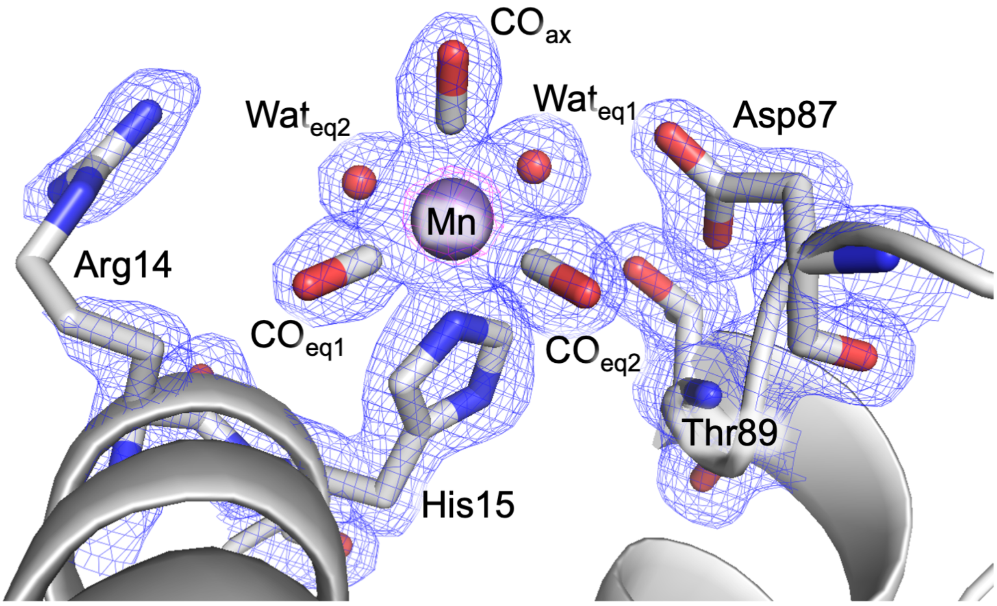

Supplementary Figure 1: The structure of HEWL-Mn(CO)$_3$(wat)$_2$ determined from a single crystal at 100K. The 2F_o-F_c map at 1σ and anomalous map at 6σ are shown in blue and magenta mesh, respectively.

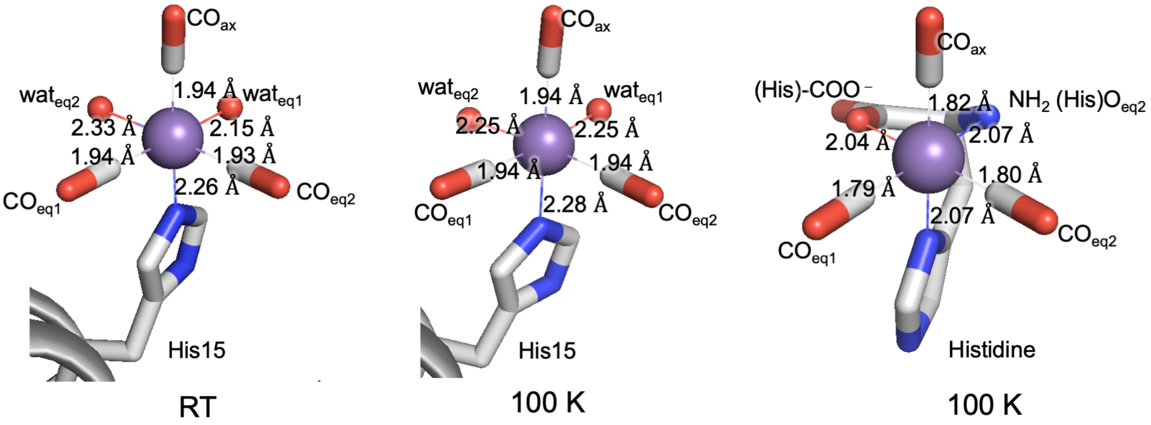

Supplementary Figure 2: Comparison of the coordination structure of Mn(CO)$_3$(wat)$_2$ moiety in (a) HEWL-Mn(CO)$_3$(wat)$_2$ at RT (b) HEWL-Mn(CO)$_3$(wat)$_2$ at 100 K and (c) Histidine-Mn(CO)$_3$ at 100 K. Color codes are as follows: Carbon: grey; Nitrogen: blue; Oxygen: red; Manganese: purple. Microcrystals were used for RT data collection by serial crystallography.

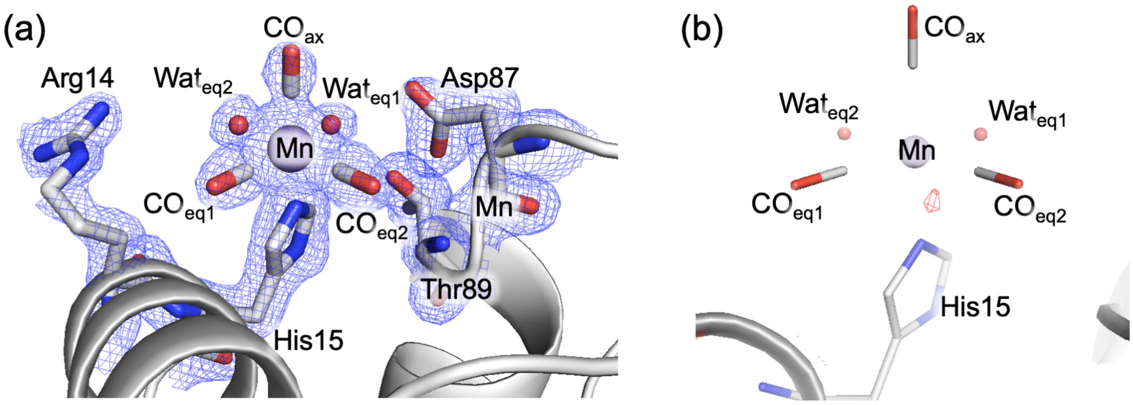

Supplementary Figure 3: (a) The structure of HEWL-$Mn(CO)_3(wat)_2$ under darkness. The structure was collected with an interval of 24h time to see the possibility of any dark reaction in comparison to the darkness structure shown in the Figure 3b in main text. The $2F_o$-F_c map contoured at 1σ is shown blue mesh. (b) The F_{data2}-F_{data1} map (F_o-F_o) contoured at ±3.2 σ is shown in green (positive) and red (negative). The data were collected under dark conditions at RT by SFX.

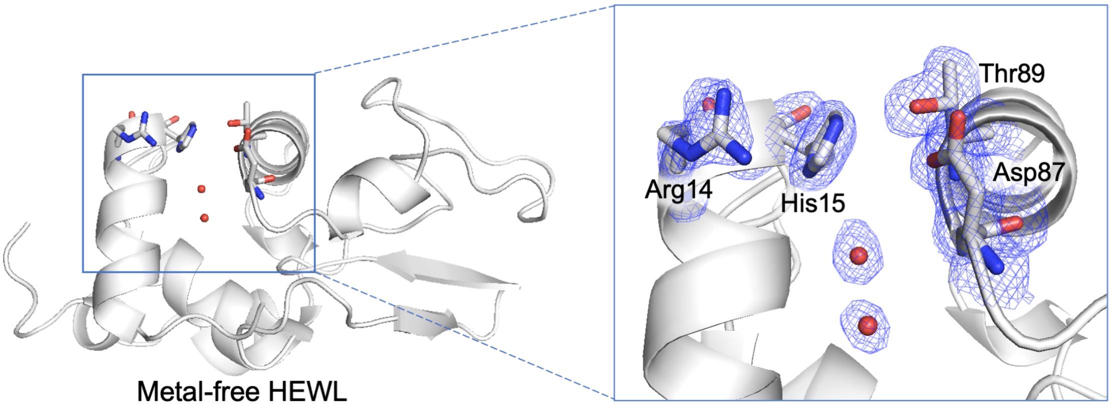

Supplementary Figure 4: Crystal structure of a metal-free HEWL structure as determined by SFX. The metal binding site at His15 and the surrounding Arg14, Asp87 and Thr89 residues are highlighted by stick model. The $2F_o$-F_c electron density map surrounding the Arg14, His15, Asp87 and Thr89 are shown in blue mesh contoured at 1.0 σ.

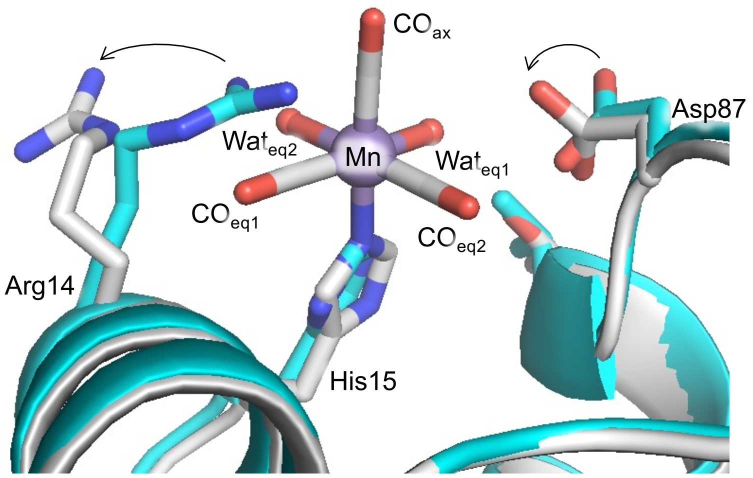

Supplementary Figure 5: Structural overlap of the metal-free HEWL (cyan) and HEWL-$Mn(CO)_3(wat)_2$ (grey) structures showing the conformational changes of Arg14 and Asp87 after metal binding. Color code for metal-free HEWL: Cartoon, cyan; carbon, cyan; nitrogen, blue; oxygen, red. Color code for HEWL-$Mn(CO)_3(wat)_2$: Cartoon, grey; carbon, grey; nitrogen, blue; oxygen, red; Manganese, purple.

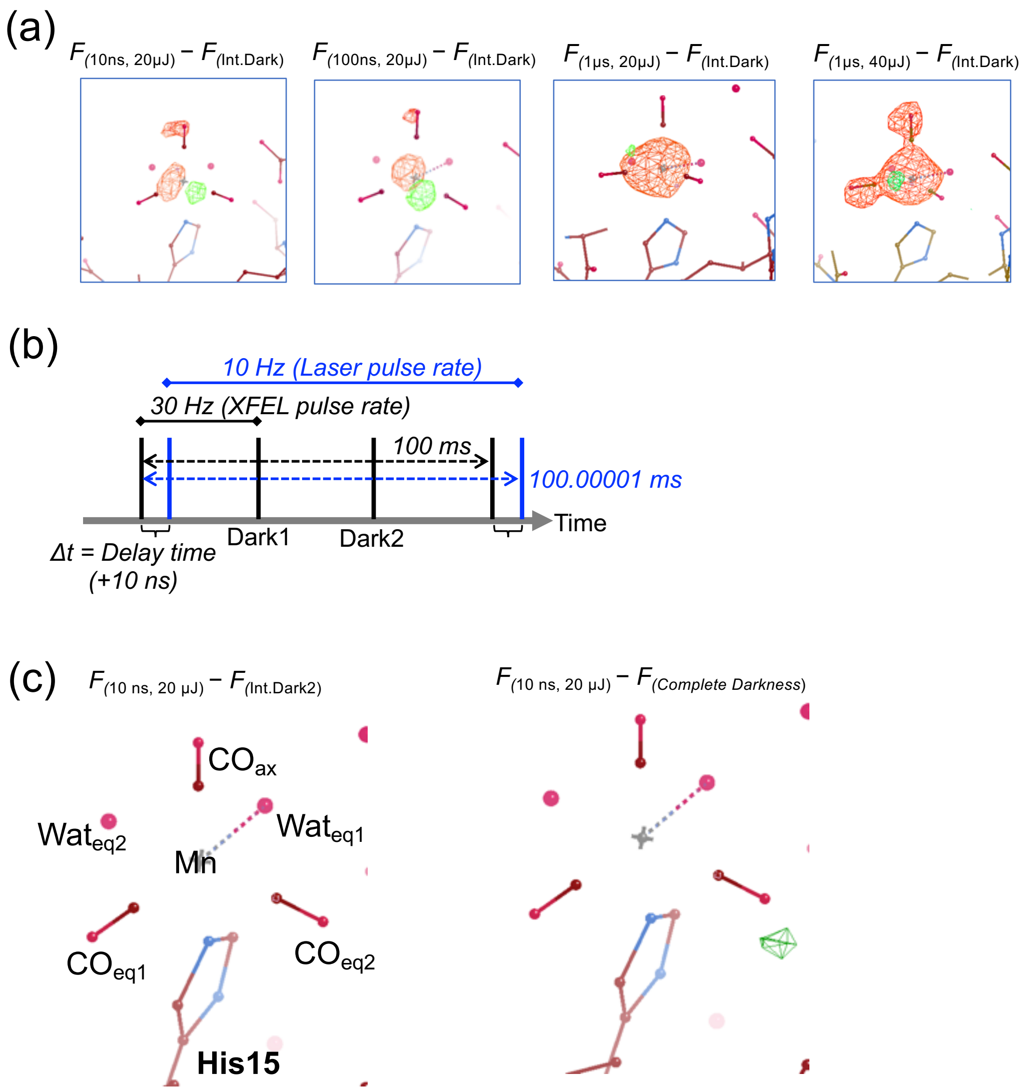

Supplementary Figure 6: (a) The difference F_{light}-F_{dark} map (at ±3.0σ, green/red) with respect to the respective interleaved dark structure showing the ligand release during the reaction. (b) A typical setup for the measurement by fixing negative delay (+10 ns) in which light is irradiated after X-ray diffraction to check any possibility of light contamination. (c) The difference density features (at 3.0 σ) on Mn-carbonyl in the negative delay experiment. No difference density feature indicates no light contamination.

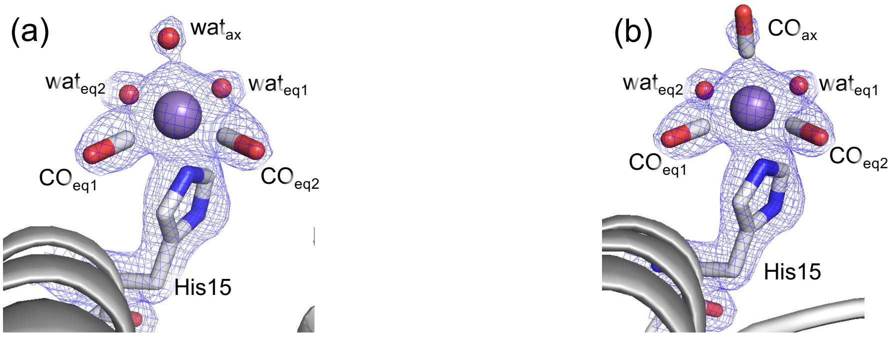

Model: HEWL-Mn$(CO)_2(wat)_3$

atom	occupancy	B-factor
**Mn**	0.9	31.12
**His15, NE2**	1	31.53
**wat$_{ax}$, O**	0.9	35.12
**CO$_{eq1}$, C**	0.9	29.98
**CO$_{eq1}$, O**	0.9	36.60
**CO$_{eq2}$, C**	0.9	31.70
**CO$_{eq2}$, O**	0.9	34.23
**Wat$_{eq1}$,O**	0.9	33.83
**Wat$_{eq2}$,O**	0.9	39.51
**R-work (R-free)**	18.57 (20.07)	

Model: HEWL-Mn$(CO)_3(wat)_2$

atom	occupancy	B-factor
**Mn**	0.9	31.45
**His15, NE2**	1	31.05
**CO$_{ax}$, C**	0.9	37.41
**CO$_{ax}$, O**	0.9	45.10
**CO$_{eq1}$, C**	0.9	30.65
**CO$_{eq1}$, O**	0.9	37.26
**CO$_{eq2}$, C**	0.9	32.52
**CO$_{eq2}$, O**	0.9	34.73
**Wat$_{eq1}$,O**	0.9	33.48
**Wat$_{eq2}$,O**	0.9	39.85
**R-work (R-free)**	18.50 (20.03)	

Supplementary Figure 7: Assignment of the axial ligand in the HEWL-Mn$(CO)_3(wat)_2$ structure, 10 ns after the photoexcitation (Laser intensity 20 uJ). (a) Occupancy and B-factors (Å^2) of selected atoms when the axial ligand is assigned to a water molecule. (b) Occupancy and B-factors (Å^2) when the axial ligand is assigned to CO ligand. The $2F_o$-F_c maps in both images are contoured at 1.2σ and shown as blue mesh. The high B-factor and spherical $2F_o$-F_c map density of the axial ligand in (b) suggest that the assignment of a water molecule for the axial ligand in (a) might be more accurate.

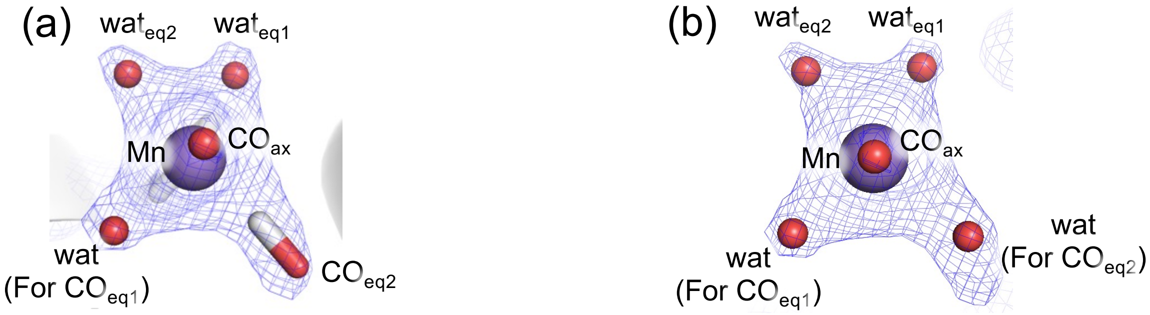

Model: HEWL-Mn(CO)(wat)$_4$

atom	occupancy	B-factor
**Mn**	0.65	44.14
**His15, NE2**	1.0	45.60
**Wat$_{ax}$**	0.65	46.85
**Wat (for CO$_{eq1}$)**	0.65	45.53
**CO$_{eq2}$, C**	0.65	40.91
**CO$_{eq2}$, O**	0.65	44.45
**Wat$_{eq1}$, O**	0.65	48.50
**Wat$_{eq2}$, O**	0.65	52.09
**R-work (R$_{free}$)**	0.1902 (0.1913)	

Model: HEWL-Mn(wat)$_5$

atom	occupancy	B-factor
**Mn**	0.65	43.9
**His15, NE2**	1.0	44.3
**Wat$_{ax}$**	0.65	44.7
**Wat (for CO$_{eq1}$)**	0.65	44.6
**Wat (for CO$_{eq2}$)**	0.65	41.4
**Wat$_{eq1}$, O**	0.65	45.9
**Wat$_{eq2}$, O**	0.65	49.7
**R-work (R$_{free}$)**	0.1903 (0.1987)	

Supplementary Figure 8: Assignment of the equatorial (eq2) ligand in the HEWL-Mn(CO)$_3$(wat)$_2$ structure, 1µs after the photoexcitation (Laser intensity 40 uJ). (a) Occupancy and B-factors (Å^2) of selected atoms when the eq2 ligand is assigned to a CO ligand. (b) Occupancy and B-factors (Å^2) when the eq2 ligand is assigned to a water. The $2F_o$-F_c map contoured at 1σ is shown in blue mesh in both figures (view from CO$_{ax}$). Considering the $2F_o$-F_c , F_o-F_c and omit map shape at eq2 position, assignment of a CO ligand might be more accurate than a water.

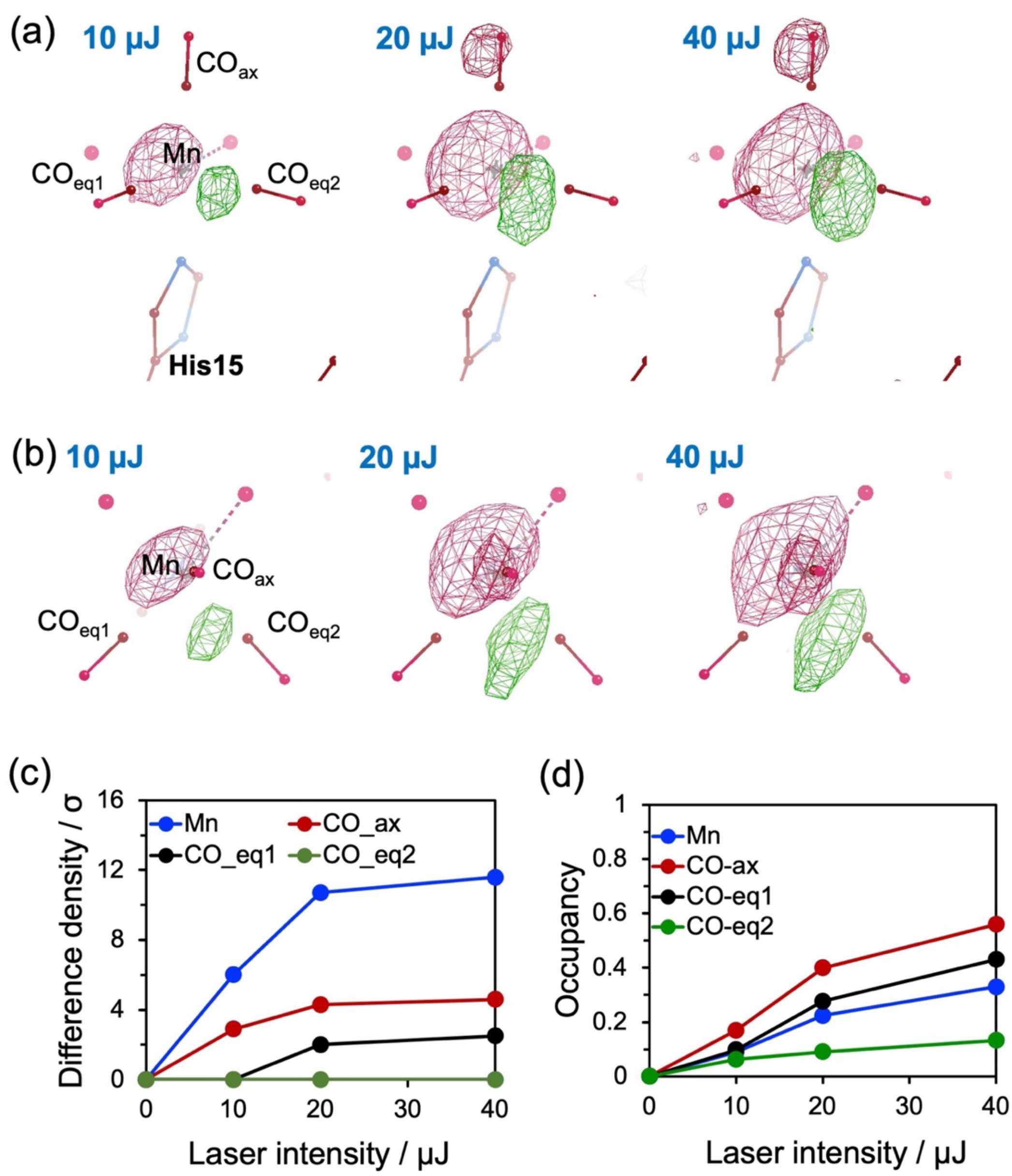

Supplementary Figure 9A: Power titration of the CO release reaction in HEWL-Mn(CO)$_3$ for 10 ns delay. The data presented are from an independent experiment measured at the same beamtime. (a) The difference $|F_{obs}|^{light}$ -$|F_{obs}|^{comp.dark}$ map (at ±3.2σ, green/red) with respect to the structure under darkness showing the ligand release during the reaction at laser intensity 10 µJ, 20 µJ and 40 µJ. The maps are shown on the model under complete darkness. (b) View of the difference $|F_{obs}|^{light}$ -$|F_{obs}|^{comp.dark}$ map from the direction of CO$_{ax}$ corresponding to the images as shown in (a). (c) Plot of difference density feature (negative) vs laser intensity to show the effect of laser intensity on CO release reaction. The density feature ($|F_{obs}|^{light}$ -$|F_{obs}|^{comp.dark}$) below 2.0σ are considered insignificant due to high signal to noise ratio. (d) Plot showing the apparent occupancy vs laser intensity. The apparent occupancy of the released CO or Mn was calculated based on the respective omit map density in dark and light irradiated state using the formula: Occupancy = (dark state-light state)/dark state.

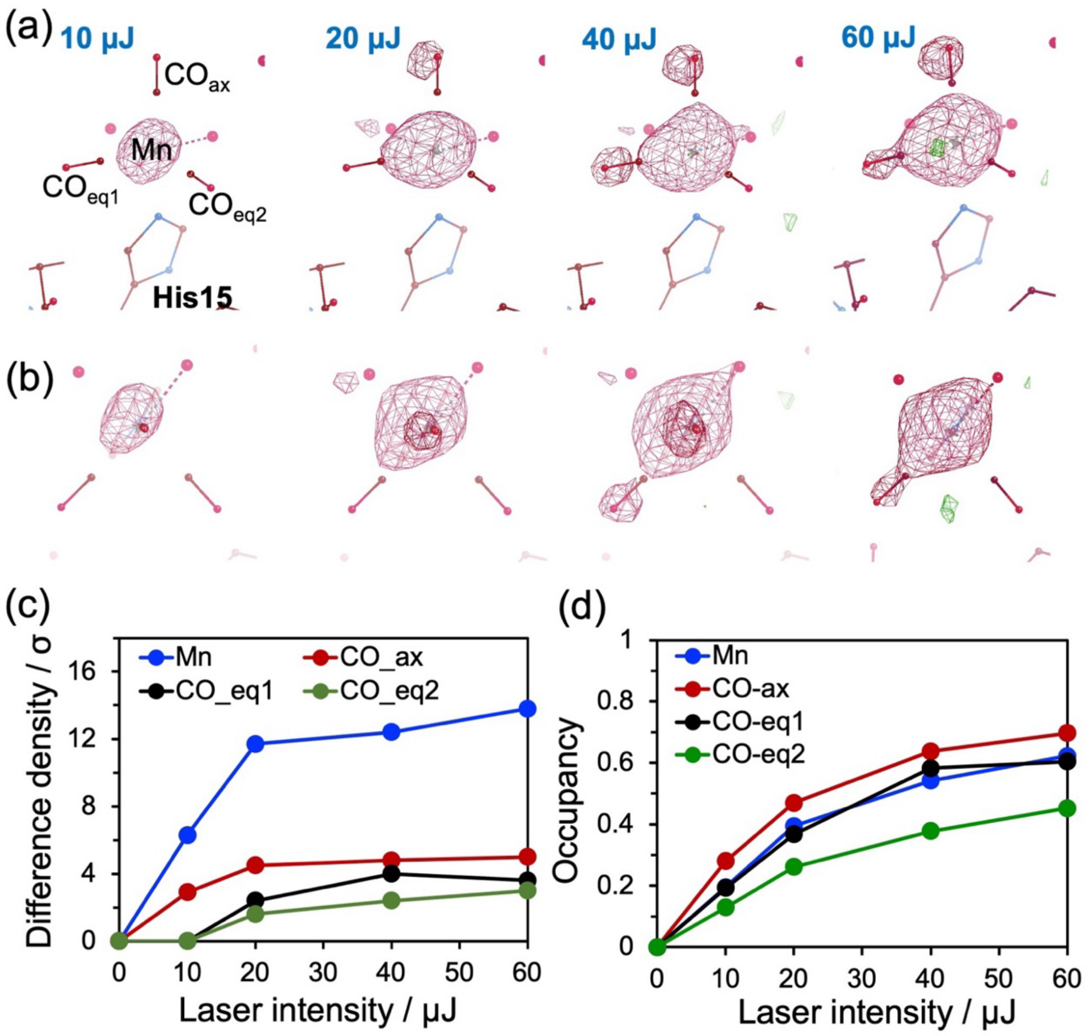

Supplementary Figure 9B: Power titration of the CO release reaction in HEWL-Mn(CO)$_3$ for 1 μs delay. The data presented are from an independent experiment measured at the same beamtime. (a) The difference $|F_{obs}|^{light}$ -$|F_{obs}|^{comp.dark}$ map (at ±3.2σ, green/red) with respect to structure under darkness showing the ligand release during the reaction at laser intensity 10 μJ, 20 μJ and 40 μJ. The maps are shown on the model under complete darkness. (b) View of the difference $|F_{obs}|^{light}$ - $|F_{obs}|^{comp.dark}$ map from the direction of CO$_{ax}$ corresponding to the images as shown in (a). (c) Plot of difference density feature (negative) vs laser intensity to show the effect of laser intensity on CO release reaction. The density feature ($|F_{obs}|^{light}$ -$|F_{obs}|^{comp.dark}$) below 2.0σ are considered insignificant due to high signal to noise ratio. (d) Plot showing the apparent occupancy vs laser intensity. The apparent occupancy of the released CO or Mn was calculated based on the respective omit map density in dark and light irradiated state using the formula: Occupancy = (dark state-light state)/dark state.

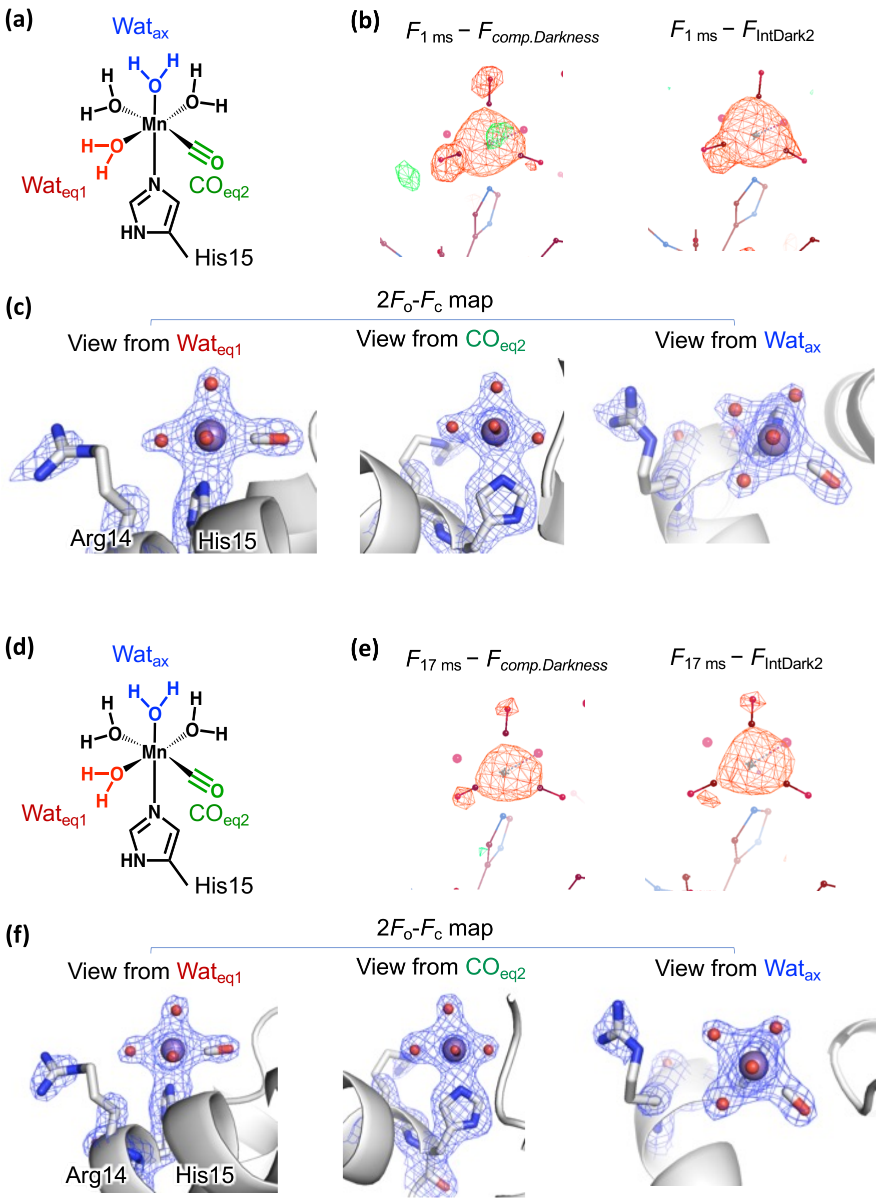

Supplementary Figure 10: Analysis of HEWL-$Mn(CO)_3(wat)_2$ structure at longer delay after the photoexcitation. (a) Coordination structure of HEWL-$Mn(CO)_3(wat)_2$ after 1ms of photoexcitation with 20 µJ laser intensity. (b) The difference F_{light}-F_{dark} map (at ±3.0σ, green/red) against complete dark and the difference F_{light}-F_{dark} map (at ±3.0σ, green/red) against interleaved dark2 structure for 1ms delay time. The maps are shown on the dark model structure. (c) $2F_o$-F_c map (at 1σ) for the model after 1 ms of photoexcitation. (d) Coordination structure of HEWL-$Mn(CO)_3(wat)_2$ after 17 ms of photoexcitation. (e) The difference F_{light}-F_{dark} map (at ±3.0σ, green/red) and the difference F_{light}-F_{dark} map (at ±3.0σ, green/red) against complete darkness and interleaved dark2 structure, respectively, for 17 ms delay time. The maps are shown on the dark model structure. (f) $2F_o$-F_c map (at 1σ) for the model after 17 ms of photoexcitation.

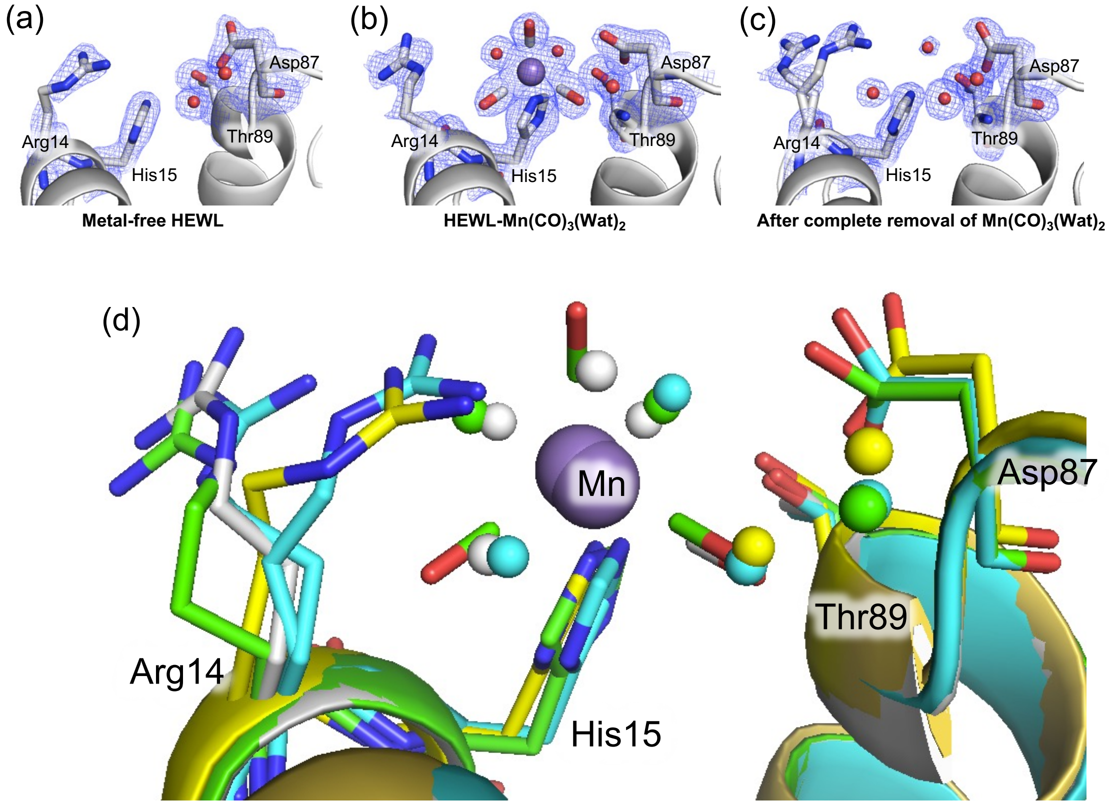

Supplementary Figure 11: Changes in the side chain residues during the CO release reaction. (a-c) Comparison of the structures of metal-free HEWL, HEWL-$Mn(CO)_3(Wat)_2$ and after complete removal of $Mn(CO)_3(Wat)_2$. Complete removal of $Mn(CO)_3(wat)_2$ was achieved by irradiating a 8W UV lamp (365nm) onto a single crystal for 20min followed by X-ray diffraction measurement at 100K in Rigaku XtalLab Synergy diffractometer. $2F_o$-F_c maps (at 1σ) are shown in blue mesh. (d) Overlap of the structure of metal-free HEWL (yellow), $Mn(CO)_3$ bounded under darkness (green), after 1μs (40μJ) of photoexcitation (grey) and after complete release of CO (cyan).

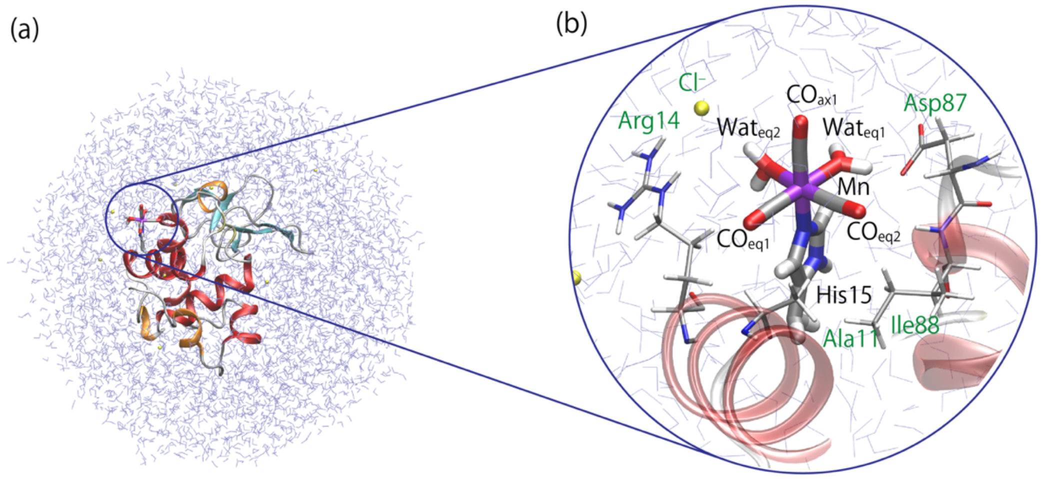

Supplementary Figure 12. Entire QM/MM system used in the present study. (a) Arrangement of HEWL in a water droplet. (b) Enlarged view for the $Mn(CO)_3(Wat)_2$ center in HEWL. Atoms in the QM region are shown in thick licorice representation.

(A)

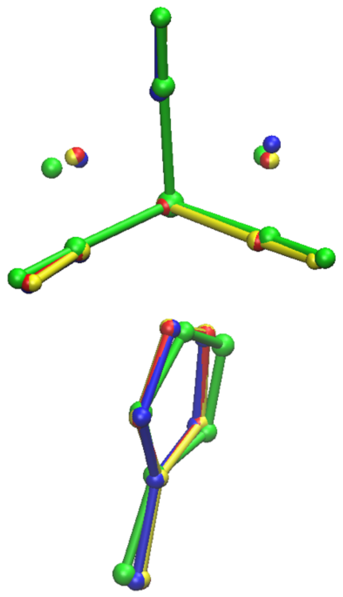

(B)

	B3LYP//DZVP[1] (B1)	B3LYP//TZVP[2] (B2)	B3LYP//TZVP+[3] (B3)	SFX (8WZF)
B1	0	0.088	0.096	0.228
B2	0.088	0	0.029	0.216
B3	0.096	0.029	0	0.216
X1	0.228	0.216	0.216	0

[1]DZVP: LANL2DZ for Mn, 6-31G* for other atoms

[2]TZVP: LANL2TZ for Mn, 6-311G* for other atoms

[3]TZVP+: LANL2TZ for Mn, 6-311+G* for other atoms

Supplementary Figure 13. (A) Superimposed structures of the Mn center by using different QM/MM method and the SFX method. Atoms are drawn with different colors: Blue for B1, Red for B2, Yellow for B3, Green for SFX. (B) Comparison of root-mean-square deviation of heavy atomic positions (RMSD) for the QM/MM optimized structures and that of the SFX crystal structure (PDBID:8WZF) in the state **1**. Different basis sets are adapted for the QM/MM optimizations. RMSD values are given in Angstrom(Å).

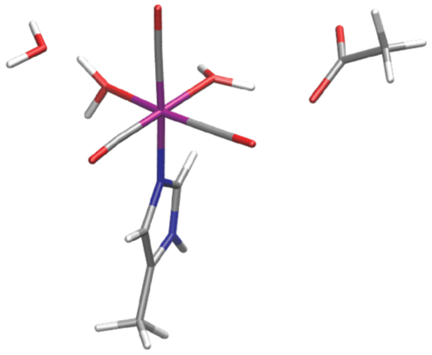

(**1**)

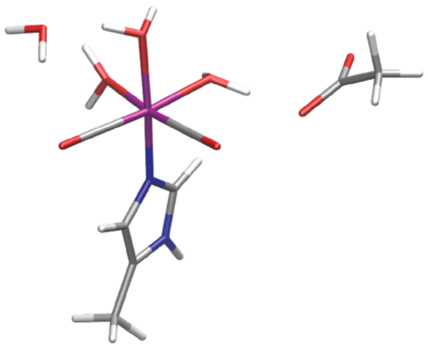

(**2**)

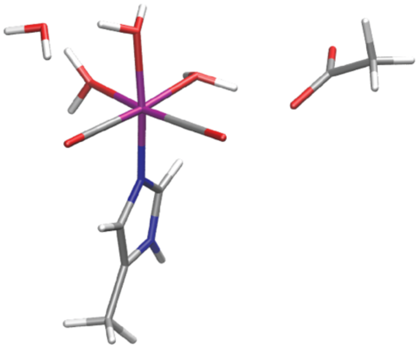

(**3**)

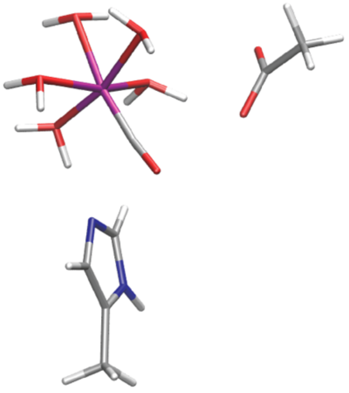

(**4**)

Supplementary Figure 14. Enlarged view of the QM region in the QM/MM calculations for the calculations of UV-spectra. TDDFT calculations were performed at the CAMB3LYP/TZVP+ level of theory. Calculated UV spectra were shown in Figure 5. The Asp 87 side chain was explicitly included in the QM region for the calculations of UV spectra. However, the Asp87 side chain was included in the MM region in the calculations of reaction paths to reduce the computational cost.

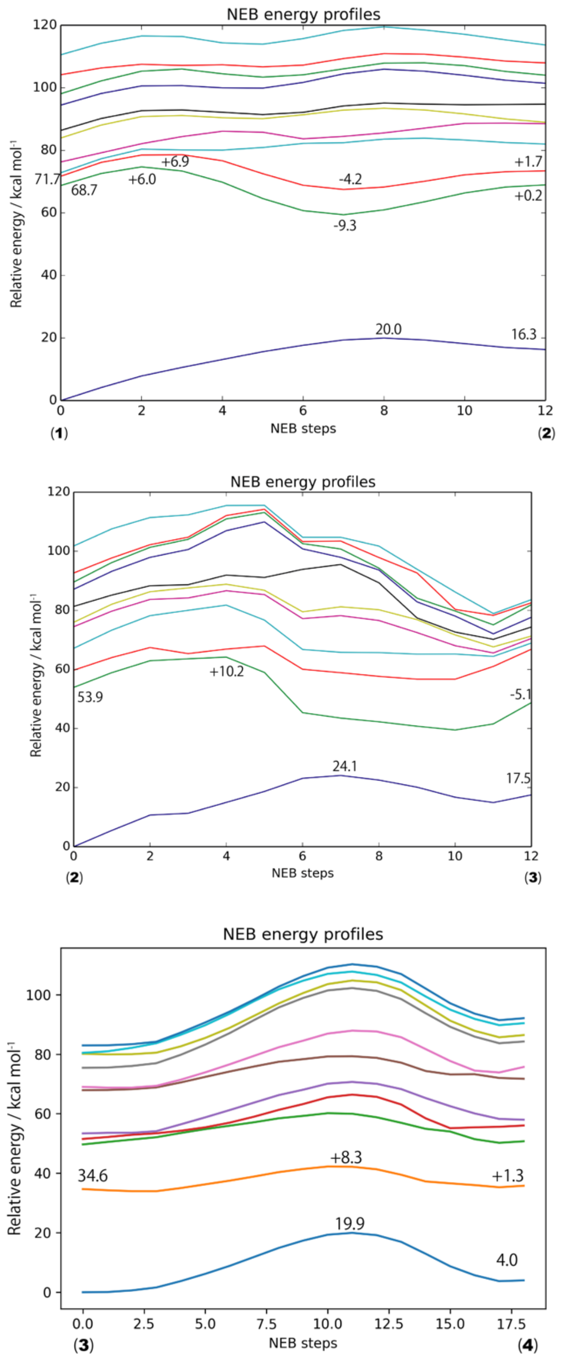

Supplementary Figure 15. NEB energy profiles along the main ligand-dissociation reactions. Relative energies are given in kcal mol^{-1}.

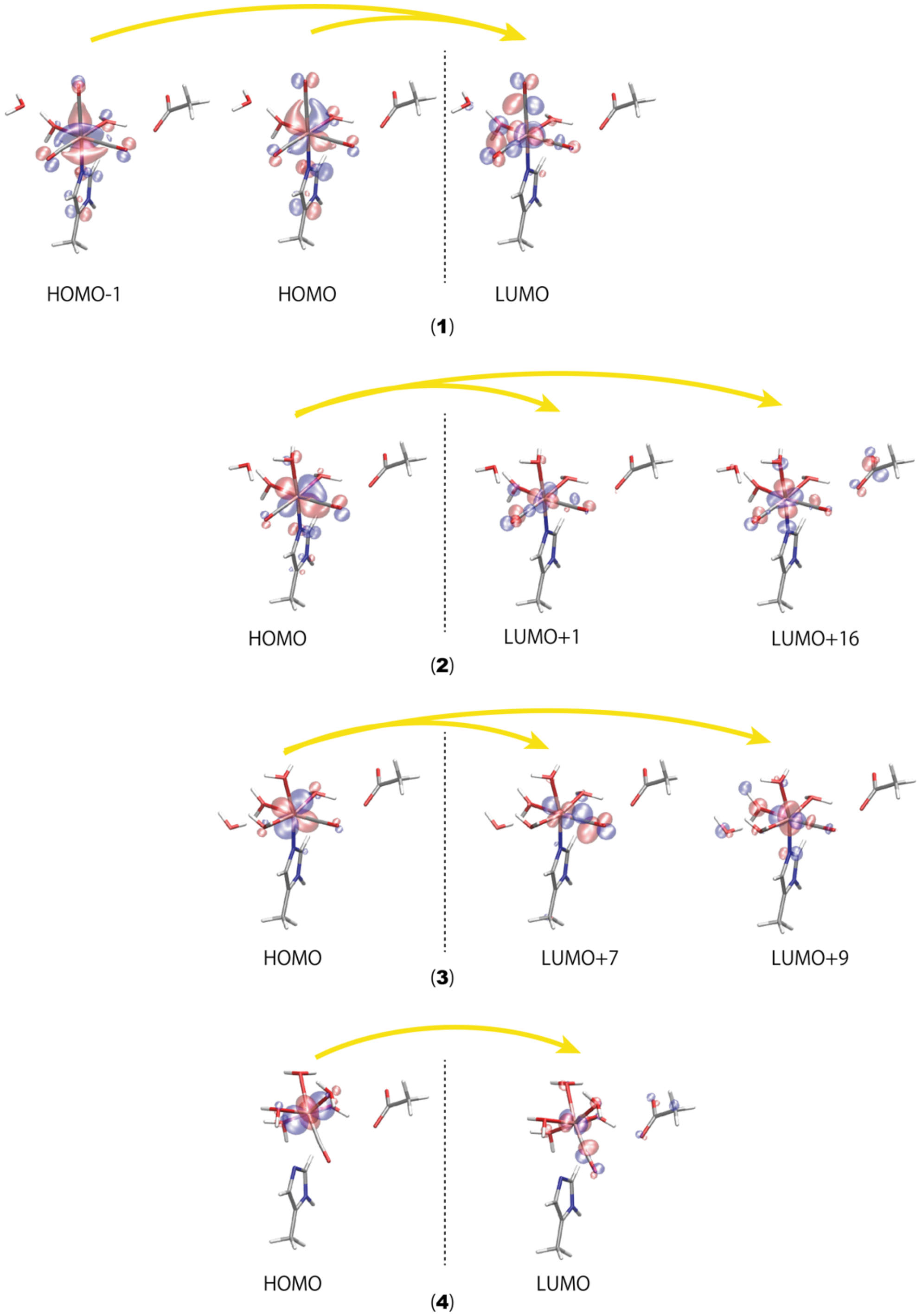

Supplementary Figure 16. Molecular orbitals mainly contribute to the first excited state calculated under the CAMB3LYP/TZVP+ level of theory. Atoms in the QM region of the QM/MM calculations are shown in Supplementary Figure 15. These HOMOs are mostly distributed on the Mn d orbitals, while the LUMOs are more delocalized to the CO and water ligands with antibonding character. These MO transitions support the ligand dissociation in these excited states.

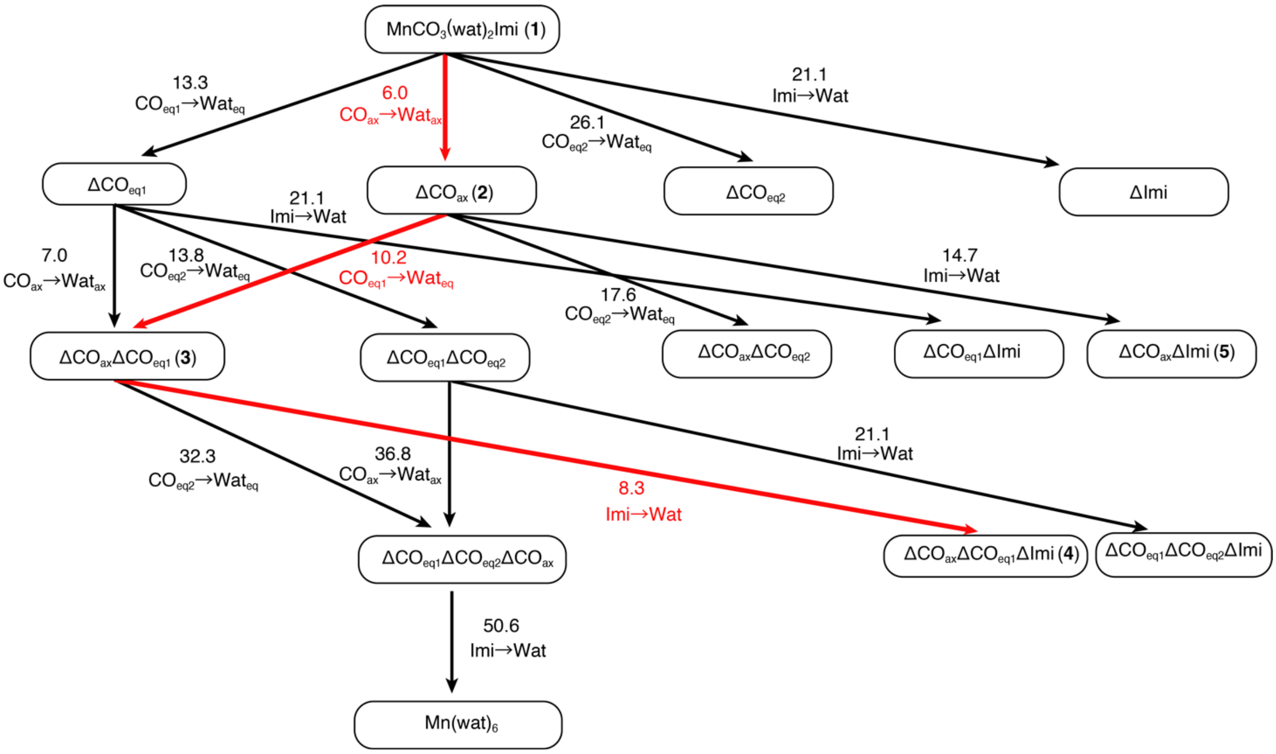

Supplementary Figure 17: Schematic representation showing the flow chart of all possible CO release steps with the energy barrier values in kcal mol^{-1} units. The corresponding ground state and excited state energies are listed in Supplementary Table 8.

Supplementary Table 1A: Crystallographic data and refinement statistics for HEWL-$Mn(CO)_3$ structures under darkness and illuminated conditions.[a]

**Dataset**	**Complete Darkness**	**10 ns 20 μJ**	**100 ns 20 μJ**	**1 μs 20 μJ**	**1 μs 40 μJ**
**PDB code**	8WZF	8WZG	8WZR	8WZT	8WZV
Data-collection date	2020/11/24	2020/11/24	2021/11/02	2021/11/02	2022/10/25
Wavelength (Å)	1.0	1.0	1.0	1.0	1.0
Space group	$P4_32_12$	$P4_32_12$	$P4_32_12$	$P4_32_12$	$P4_32_12$
Hit images	15663	16172	23627	23656	20096
Indexed images	14714	15575	22963	22882	17709
Unit cell *a, b, c* (Å); α, β, ν (°)	81.0, 81.0, 37.7; 90.0, 90.0, 90.0	81.0, 81.0, 37.7; 90.0, 90.0, 90.0	81.7, 81.7, 37.9; 90.0, 90.0, 90.0	81.7, 81.7, 37.7; 90.0, 90.0, 90.0	80.8, 80.8, 37.6; 90.0, 90.0, 90.0
Resolution range (Å)	31.44-1.60 (1.62-1.60)	31.44-1.60 (1.62-1.60)	31.64-1.60 (1.62-1.60)	31.64-1.60 (1.62-1.60)	31.44-1.60 (1.62-1.60)
Total reflections	7680482 (192494)	8081709 (190265)	14374496 (205062)	13068826 (244841)	8214252 (74168)
Unique reflections	17136 (841)	17136 (841)	17528 (859)	17528 (859)	17017 (842)
Multiplicity	448.2 (228.9)	471.6 (226.2)	820.1 (238.7)	745.6 (285.0)	482.7 (88.1)
Completeness (%)	100.0 (100.0)	100.0 (100.0)	100.0 (100.0)	100.0 (100.0)	100.0 (100.0)
Mean I/sigma (I)	6.82 (4.18)	8.04 (4.21)	9.55 (1.18)	8.54 (1.06)	8.20 (0.92)

Wilson B-factor (Å^2)	25.16	25.51	36.82	38.11	35.8
R-split	14.0 (22.7)	11.1 (21.3)	8.2 (86.4)	9.2 (90.6)	9.4 (113.8)
$CC_{1/2}$	0.965 (0.906)	0.980 (0.934)	0.990 (0.486)	0.987 (0.537)	0.987 (0.391)
Reflections used in refinement	17094	17093	17481	17483	16969
Reflections used for R-free	855	855	875	875	849
R-work (%)	19.30	18.52	19.48	19.27	19.02
R-free (%)	22.63	20.05	19.95	21.23	19.12
Number of non-hydrogen atoms	1093	1091	1095	1093	1068
Macromolecules	998	990	1020	1017	993
Ligands	9	10	7	8	5
Solvent	86	91	68	68	70
Protein residues	129	129	129	129	129
RMS deviation (bonds, Å)	0.007	0.006	0.009	0.008	0.011
RMS deviation (angles, °)	0.94	0.96	1.09	1.14	1.64
Ramachandran favored (%)	99.21	99.21	97.64	99.21	99.21
Ramachandran allowed (%)	0.79	0.79	2.36	0.79	0.79
Ramachandran outliers (%)	0	0	0	0	0

Rotamer outliers (%)	0	0	0.91	0.92	0.96
Clash score	2.03	1.03	5.44	1.49	2.05
Average B-factor ($Å^2$)	22.50	24.19	33.46	34.02	35.71
macromolecules ($Å^2$)	21.61	23.13	32.86	33.38	35.06
ligands ($Å^2$)	33.27	34.17	40.65	42.68	41.48
Solvent ($Å^2$)	31.71	34.66	41.79	42.63	44.53

Note: Values in the parentheses are for the highest-resolution shell. $R = \Sigma||F_o|-|F_c||/\Sigma|F_o|$, where F_o and F_c are the observed and calculated structure factor amplitudes, respectively. R_{free}: The R factor calculated on a partial set that is not used in the refinement of the structure. Ramachandran plot parameters were obtained from Molprobity.

[a]The data used in Figure 4c in the main text.

Supplementary Table 1B: Crystallographic data and refinement statistics for HEWL-$Mn(CO)_3$ structures under dark conditions.

**Dataset**	**Complete Darkness**[a]	**10 ns 20 µJ-dark2**[b]	**Complete Darkness**[c]	**100 ns 20 µJ-Dark**[d]	**1 µs 20 µJ-Dark**[e]	**Metal-free HEWL**
Data collection date	2020/11/24	2020/11/24	2021/11/02	2021/11/02	2021/11/02	2021/11/02
Wavelength (Å)	1.0	1.0	1.0	1.0	1.0	1.0
Space group	$P4_32_12$	$P4_32_12$	$P4_32_12$	$P4_32_12$	$P4_32_12$	$P4_32_12$
Hit images	33166	16903	29920	25487	25583	29731
Indexed images	30384	15968	27755	23435	23977	24491
Unit cell *a* , *b* , *c* (Å); α , β , *ν* (°)	81.0, 81.0, 37.7; 90.0, 90.0, 90.0	81.0, 81.0, 37.7; 90.0, 90.0, 90.0	81.7, 81.7, 37.9; 90.0, 90.0, 90.0	81.7, 81.7, 37.9; 90.0, 90.0, 90.0	81.7, 81.7, 37.9; 90.0, 90.0, 90.0	79.2, 79.2, 38.1; 90.0, 90.0, 90.0
Resolution range (Å)	31.44-1.60 (1.62-1.60)	31.44-1.60 (1.62-1.60)	31.64-1.60 (1.62-1.60)	31.64-1.60 (1.62-1.60)	31.44-1.60 (1.62-1.60)	31.54-1.60 (1.62-1.60)
Total reflections	14501712 (361248)	7942477 (192001)	15230346 (369011)	13810716 (355727)	14466062 (370389)	18189568 (517185)
Unique reflections	17136 (841)	17136 (841)	17528 (859)	17528 (859)	17528 (859)	16564 (799)
Multiplicity	846.3 (429.5)	463.5 (228.3)	868.9 (429.6)	787.9 (414.1)	825.3 (431.2)	1098.1 (647.3)
Completeness (%)	100.0 (100.0)	100.0 (100.0)	100.0 (100.0)	100.0 (100.0)	100.0 (100.0)	100.0 (100.0)
Mean I/sigma (I)	9.59 (5.57)	7.40 (4.31)	8.44 (1.41)	7.78 (1.71)	87.92 (1.59)	9.19 (3.41)
Wilson B-factor ($Å^2$)	25.21	24.77	37.14	34.74	35.2	30.69
R-split	9.50 (17.43)	12.58 (23.14)	10.27 (69.85)	11.08 (56.16)	11.04(60.48)	10.63 (28.80)
$CC_{1/2}$	0.9841 (0.9235)	0.9718 (0.8813)	0.9816 (0.6306)	0.9807(0.7056)	0.9795 (0.7003)	0.98 (0.59)

Reflections used in refinement	17094	17094	17484	17484	17485	12375
Reflections used for R-free	1220	855	875	875	875	620
R-work (%)	19.12	19.17	20.60	19.85	19.72	19.09
R-free (%)	20.75	19.94	21.58	22.43	20.40	23.10
Number of non-hydrogen atoms	1091	1094	1079	1096	1084	1082
Macromolecules	998	997	1001	1000	991	1004
Ligands	10	10	9	9	11	2
Solvent	83	87	69	87	82	76
Protein residues	129	129	129	129	129	129
RMS deviation (bonds, Å)	0.008	0.007	0.012	0.010	0.007	0.010
RMS deviation (angles, °)	0.99	1.02	1.45	1.15	0.87	0.92
Ramachandran favored (%)	99.21	99.21	97.64	99.21	98.43	99.21
Ramachandran allowed (%)	0.79	0.79	2.36	0.79	1.57	0.79
Ramachandran outliers (%)	0	0	0	0	0	0
Rotamer outliers (%)	0	0	0	0	0	0
Clash score	4.07	3.06	1.52	0.51	1.02	1.52
Average B-factor (Å^2)	22.13	22.56	31.08	29.33	29.50	19.48
Macromolecules (Å^2)	21.26	21.63	30.43	28.46	28.62	18.89

ligands (Å^2)	31.65	32.71	40.57	38.66	39.71	22.67
Solvent (Å^2)	31.52	32.06	39.30	38.31	38.77	27.19

Note: Values in the parentheses are for the highest-resolution shell. $R = \Sigma||F_o|-|F_c||/\Sigma|F_o|$, where F_o and F_c are the observed and calculated structure factor amplitudes, respectively. R_{free}: The R factor calculated on a partial set that is not used in the refinement of the structure. Ramachandran plot parameters were obtained from Molprobity.
[a]A complete darkness data used in Supplementary Figure 3 in SI. This darkness structure was collected with an interval of over 24h time to see the possibility of any dark reaction in comparison to the structure shown in Figure 3b in the main text.
[b]Interleaved dark dataset 2 for 10ns20μJ collected with 10Hz repetition.
[c,d,e]Complete darkness and interleaved dark data corresponding to datasets of delay time 100ns and 1μs as shown in Figure 4c-iii,iv. The light data were collected with 15Hz repetition.

Supplementary Table 1C: Crystallographic data and refinement statistics for HEWL-Mn(CO)$_3$ structures under selected conditions.[a]

**Dataset**	**Complete Darkness**	**1µs40µJ-dark2**	**1ms20µJ-light**	**1 ms20 µJ_Dark2**	**17ms20 µJ-light**	**17ms20 µJ-dark2**
Data collection date	2022/10/25	2022/10/25	2022/10/25	2022/10/25	2022/10/25	2022/10/25
Wavelength (Å)	1.0	1.0	1.0	1.0	1.0	1.0
Space group	$P4_32_12$	$P4_32_12$	$P4_32_12$	$P4_32_12$	$P4_32_12$	$P4_32_12$
Hit images	26248	25001	18547	20958	29107	31290
Indexed images	22498	20229	16909	18681	20733	21902
Unit cell *a* , *b* , *c* (Å); α , β, *ν* (°)	80.8, 80.8, 37.6; 90.0, 90.0, 90.0	80.8, 80.8, 37.6; 90.0, 90.0, 90.0	80.8, 80.8, 37.6; 90.0, 90.0, 90.0	80.8, 80.8, 37.6; 90.0, 90.0, 90.0	80.8, 80.8, 37.6; 90.0, 90.0, 90.0	80.8, 80.8, 37.6; 90.0, 90.0, 90.0
Resolution range (Å)	31.44-1.60 (1.62-1.60)	31.44-1.60 (1.62-1.60)	31.64-1.60 (1.62-1.60)	31.64-1.60 (1.62-1.60)	31.44-1.60 (1.62-1.60)	31.44-1.60 (1.62-1.60)
Total reflections	9906473 (250199)	8487553 (204454)	8324674 (125815)	7761093 (191375)	8268446 (131678)	8242297 (179195)
Unique reflections	17017 (842)	17017 (842)	17017 (842)	17017 (842)	17017 (842)	17017 (842)
Multiplicity	582.2 (297.1)	498.8 (242.8)	489.2 (149.4)	456.1 (227.3)	485.9 (156.4)	484.4 (212.8)
Completeness (%)	100.0 (100.0)	100.0 (100.0)	100.0 (100.0)	100.0 (100.0)	100.0 (100.0)	100.0 (100.0)
Mean I/sigma (I)	9.04 (3.05)	8.33 (2.48)	8.17 (1.69)	8.14 (2.50)	8.54 (1.72)	8.52 (2.17)
Wilson B-factor (Å^2)	31.83	32.49	32.57	32.09	33.07	32.63
R-split	10.3 (31.6)	11.2 (36.6)	10.7 (52.3)	11.3 (37.5)	10.4 (55.8)	10.8 (43.9)

$CC_{1/2}$	0.981 (0.854)	0.978 (0.839)	0.982 (0.729)	0.978 (0.821)	0.981 (0.694)	0.978 (0.799)
Reflections used in refinement	16973	16972	16971	16971	16972	16971
Reflections used for R-free	849	849	849	849	849	849
R-work (%)	19.28	19.07	18.81	19.05	18.48	18.85
R-free (%)	21.59	21.67	20.26	22.07	20.61	21.51
Number of non-hydrogen atoms	1089	1093	1095	1092	1105	1090
Macromolecules	1000	1012	1011	1012	1017	1000
Ligands	9	9	7	9	6	10
Solvent	80	77	77	71	70	80
Protein residues	129	129	129	129	129	129
RMS deviation (bonds, Å)	0.008	0.10	0.008	0.007	0.008	0.007
RMS deviation (angles, °)	1.02	1.30	1.09	0.98	1.09	0.97
Ramachandran favored (%)	99.21	99.21	97.64	99.21	98.43	98.43
Ramachandran allowed (%)	0.79	0.79	2.36	0.79	1.57	1.57
Ramachandran outliers (%)	0	0	0	0	0	0
Rotamer outliers (%)	0.94	0	0	0	0.92	0.94
Clash score	1.01	3.02	2.00	1.00	1.99	2.54
Average B-factor ($Å^2$)	27.53	29.37	31.37	28.72	31.13	29.72
Macromolecules ($Å^2$)	26.77	28.56	30.67	28.07	30.35	28.98

Ligands (Å^2)	36.64	39.22	36.36	39.17	38.47	39.56
Solvent (Å^2)	36.04	38.81	40.18	36.55	40.42	37.84

Note: Values in the parentheses are for the highest-resolution shell. $R = \Sigma||F_o|-|F_c||/\Sigma|F_o|$, where F_o and F_c are the observed and calculated structure factor amplitudes, respectively. R_{free}: The R factor calculated on a partial set that is not used in the refinement of the structure. Ramachandran plot parameters were obtained from Molprobity. [a]Complete darkness, interleaved dark2 and light data corresponding to datasets of delay time 1µs (40µJ), 1ms (20µJ) and 17ms (20µJ). The light data were collected with 10Hz repetition.

Supplementary Table 1D: Crystallographic data and refinement statistics for HEWL-$Mn(CO)_3$ structures under darkness and illuminated conditions.[a]

Dataset	Complete Darkness	10 ns 10 μJ-light	10 ns 10 μJ - dark2	10 ns 20 μJ -light	10 ns 20 μJ -dark2
Data collection date	2024/02/02	2024/02/02	2024/02/02	2024/02/02	2024/02/02
Wavelength (Å)	1.0	1.0	1.0	1.0	1.0
Space group	$P4_32_12$	$P4_32_12$	$P4_32_12$	$P4_32_12$	$P4_32_12$
Hit images	49363	28391	32318	28667	35894
Indexed images	44114	25120	26505	25693	29076
Unit cell	80.7, 80.7, 37.5;	80.7,80.7,37.5;	80.7,80.7,37.5;	80.7,80.7,37.5	80.7,80.7,37.5;
a, b, c (Å);	90.0, 90.0, 90.0	90.0,90.0,90.0	90.0,90.0,90.0	90.0,90.0,90.0	90.0,90.0,90.0
α , β , ν (°)					
Resolution range (Å)	40.32-1.60	40.32-1.60	40.32-1.60	40.32-1.60	40.32-1.60
	(1.62-1.60)	(1.62-1.60)	(1.62-1.60)	(1.62-1.60)	(1.62-1.60)
Total reflections	16656640 (525574)	12995801 (341995)	8436123 (250921)	16770074 (369630)	9605810 (290361)
Unique reflections	16936 (843)	16936 (843)	16936 (843)	16936 (843)	16936 (843)
Multiplicity	983.5 (623.50)	767.3 (405.70)	498.1 (297.70)	16936 (843)	567.2 (344.40)
Completeness (%)	100.0 (100.00)	100.0(100.00)	100.0 (100.0)	100.0 (100.0)	100.0 (100.0)
Mean I/sigma (I)	13.03 (6.33)	12.42 (3.50)	10.24 (4.62)	12.59 (2.58)	10.56 (4.96)
Wilson B-factor (Å^2)	27.48	27.94	27.81	28.63	27.82
R-split	7.7 (15.46)	7.5 (28.45)	10.2 (19.54)	7.0 (38.60)	10.0 (18.21)

$CC_{1/2}$	0.990 (0.9131)	0.991 (0.9008)	0.981 (0.9434)	7.0 (38.60)	0.982 (0.9455)
Reflections used in refinement	20383	16888	20380	20375	20381
Reflections used for R-free	1020	845	1019	1019	1019
R-work (%)	18.66	17.68	18.43	18.18	18.35
R-free (%)	21.32	0.1985	20.25	21.39	20.42
Number of non-hydrogen atoms	1136	1115	1131	1112	1138
Macromolecules	1012	1006	1012	1006	1012
Ligands	11	9	11	9	11
Solvent	113	100	108	97	115
Protein residues	129	129	129	129	129
RMS deviation (bonds, Å)	0.007	0.006	0.007	0.007	0.007
RMS deviation (angles, °)	1.00	0.94	0.96	0.91	0.98
Ramachandran favored (%)	99.21	99.21	99.21	99.21	99.21
Ramachandran allowed (%)	0.79	0.79	0.79	0.79	0.79
Ramachandran outliers (%)	0	0	0	0	0

Rotamer outliers (%)	0	0	0	0	0
Clash score	0.50	1.01	1.00	2.01	1.50
Average B-factor ($Å^2$)	25.74	25.36	26.62	28.38	26.70
macromolecules ($Å^2$)	24.40	24.12	25.27	27.20	25.26
ligands ($Å^2$)	36.79	38.38	38.11	41.29	38.37
Solvent ($Å^2$)	36.68	36.70	38.08	39.44	38.25

Note: Values in the parentheses are for the highest-resolution shell. $R = \Sigma||F_o|-|F_c||/\Sigma|F_o|$, where F_o and F_c are the observed and calculated structure factor amplitudes, respectively. R_{free}: The R factor calculated on a partial set that is not used in the refinement of the structure. Ramachandran plot parameters were obtained from Molprobity.

[a]The data used in Supplementary Figure 9A in SI.

Supplementary Table 1E: Crystallographic data and refinement statistics for HEWL-$Mn(CO)_3$ structures under darkness and illuminated conditions.[a]

**Dataset**	**10 ns 40 µJ-light**	**10 ns 40 µJ-dark2**	**1 µs 10 µJ-light**	**1 µs 10 µJ-dark2**	**1 µs 20 µJ-light**
Data collection date	2024/02/02	2024/02/02	2024/02/02	2024/02/02	2024/02/02
Wavelength (Å)	1.0	1.0	1.0	1.0	1.0
Space group	$P4_32_12$	$P4_32_12$	$P4_32_12$	$P4_32_12$	$P4_32_12$
Hit images	25228	33368	30958	33713	29190
Indexed images	23365	27717	21340	21635	25856
Unit cell<br>*a, b, c* (Å);<br>α, β, ν (°)	80.7,80.7,37.5;<br>90.0,90.0,90.0	80.7,80.7,37.5;<br>90.0,90.0,90.0	80.7,80.7,37.5;<br>90.0,90.0,90.0	80.7,80.7,37.5;<br>90.0,90.0,90.0	80.7,80.7,37.5;<br>90.0,90.0,90.0
Resolution range (Å)	40.32-1.60 (1.62-1.60)	40.32-1.60 (1.62-1.60)	40.32-1.60(1.62-1.60)	40.32-1.60(1.62-1.60)	40.32-1.60(1.62-1.60)
Total reflections	19749625 (417750)	10460471 (332872)	9124629 (278247)	8153584 (258037)	13734938 (399769)
Unique reflections	16936 (843)	16936 (843)	16936 (843)	16936 (843)	16936 (843)
Multiplicity	1166.1 (495.60)	617.6 (394.90)	538.8 (330.10)	16936 (843)	811.0 (474.20)
Completeness (%)	100.0 (100.0)	100.0 (100.0)	100.0 (100.0)	100.0 (100.0)	100.0 (100.00)
Mean I/sigma (I)	12.25 (1.66)	10.57 (5.28)	9.90 (3.95)	9.28 (4.92)	11.10 (2.75)
Wilson B-factor ($Å^2$)	30.96	27.31	28.65	27.33	30.84
R-split	6.2 (62.99)	9.7 (16.58)	10.0 (22.99)	11.0 (19.19)	7.7 (38.86)
$CC_{1/2}$	6.2 (62.99)	0.984 (0.9586)	0.982 (0.9270)	11.0 (19.19)	0.991 (0.5649)

Reflections used in refinement	20336	20384	20379	20379	16882
Reflections used for R-free	1019	1020	1019	1019	845
R-work (%)	18.16	18.85	18.97	19.28	18.21
R-free (%)	21.10	21.09	22.90	22.15	21.89
Number of non-hydrogen atoms	1095	1133	1117	1139	1109
Macromolecules	1006	1012	1006	1012	1006
Ligands	7	11	11	11	7
Solvent	82	110	100	116	96
Protein residues	129	129	129	129	129
RMS deviation (bonds, Å)	0.006	0.007	0.006	0.013	0.006
RMS deviation (angles, °)	0.91	0.93	0.96	1.61	0.91
Ramachandran favored (%)	98.43	99.21	99.21	98.43	99.21
Ramachandran allowed (%)	1.57	0.79	0.79	1.57	0.79
Ramachandran outliers (%)	0	0	0	0	0

Rotamer outliers (%)	0	0	0	0	0
Clash score	0.50	1.50	2.51	1.00	2.52
Average B-factor ($Å^2$)	31.25	25.57	26.59	25.43	28.36
macromolecules ($Å^2$)	30.32	24.23	25.42	24.08	27.32
ligands ($Å^2$)	40.24	36.97	40.10	35.40	35.82
Solvent ($Å^2$)	41.89	36.76	36.87	36.31	38.66

Note: Values in the parentheses are for the highest-resolution shell. $R = \Sigma||F_o|-|F_c||/\Sigma|F_o|$, where F_o and F_c are the observed and calculated structure factor amplitudes, respectively. R_{free}: The R factor calculated on a partial set that is not used in the refinement of the structure. Ramachandran plot parameters were obtained from Molprobity.

[a]The data used in Supplementary Figure 9 in SI.

Supplementary Table 1F: Crystallographic data and refinement statistics for HEWL-$Mn(CO)_3$ structures under darkness and illuminated conditions.[a]

**Dataset**	**1 µs 20 µJ-dark2**	**1 µs 40 µJ-light**	**1 µs 40 µJ-dark2**	**1 µs 60 µJ-light**	**1 µs 60 µJ-dark2**
Data collection date	2024/02/02	2024/02/02	2024/02/02	2024/02/02	2024/02/02
Wavelength (Å)	1.0	1.0	1.0	1.0	1.0
Space group	$P4_32_12$	$P4_32_12$	$P4_32_12$	$P4_32_12$	$P4_32_12$
Hit images	33052	24139	31218	27168	46021
Indexed images	27959	21650	26687	21398	36187
Unit cell *a, b, c* (Å); α, β, ν (°)	80.7,80.7,37.5; 90.0,90.0,90.0	80.7,80.7,37.5; 90.0,90.0,90.0	80.7,80.7,37.5; 90.0,90.0,90.0	80.7,80.7,37.5; 90.0,90.0,90.0	80.7,80.7,37.5; 90.0,90.0,90.0
Resolution range (Å)	40.32-1.60(1.62-1.60)	40.32-1.60(1.62-1.60)	40.32-1.60(1.62-1.60)	40.32-1.70 (1.72-1.70)	40.32-1.60 (1.62-1.60)
Total reflections	10621158 (333069)	13016895 (257223)	9672291 (294456)	12818802 (291013)	13445059 (423947)
Unique reflections	16936 (843)	16936 (843)	16936 (843)	14190 (693)	16936 (843)
Multiplicity	627.1 (395.10)	768.6 (305.10)	571.1 (349.30)	14190 (693)	793.9 (502.90)
Completeness (%)	100.0 (100.0)	100.0 (100.0)	100.0 (100.0)	100.0 (100.0)	100.0 (100.00)
Mean I/sigma (I)	10.56 (5.22)	8.91 (1.19)	9.98 (4.72)	9.89 (1.32)	793.9 (502.90)

Wilson B-factor (Å^2)	27.28	32.31	27.31	34.44	27.53
R-split	9.9 (16.84)	8.5 (81.28)	10.6 (18.64)	7.8 (79.32)	8.5 (14.71)
$CC_{1/2}$	0.980 (0.9564)	0.991(0.5155)	0.982 (0.9442)	0.992 (0.5088)	0.987 (0.9699)
Reflections used in refinement	16886	16884	16891	14138	16886
Reflections used for R-free	845	845	845	706	845
R-work (%)	18.35	18.19	19.68	19.08	18.88
R-free (%)	18.86	20.56	20.15	21.83	21.61
Number of non-hydrogen atoms	1140	1086	1130	1093	1135
Macromolecules	1012	1006	1010	1006	1012
Ligands	11	5	12	6	11
Solvent	117	75	108	81	112
Protein residues	129	129	129	129	129
RMS deviation (bonds, Å)	0.007	0.006	0.013	0.012	0.013
RMS deviation (angles, °)	0.99	1.00	1.77	1.66	1.63
Ramachandran favored (%)	99.21	98.43	97.64	98.43	98.43

Ramachandran allowed (%)	0.79	1.57	2.36	1.57	1.57
Ramachandran outliers (%)	0	0	0	0	0
Rotamer outliers (%)	0	0	0	0.94	0
Clash score	1.00	2.52	0	4.54	2.50
Average B-factor (Å^2)	23.94	32.32	21.76	32.91	22.99
macromolecules (Å^2)	22.38	31.58	20.32	32.14	21.62
ligands (Å^2)	35.21	39.19	32.70	41.37	34.17
Solvent (Å^2)	36.33	41.73	34.03	41.97	34.31

Note: Values in the parentheses are for the highest-resolution shell. $R = \Sigma||F_o|-|F_c||/\Sigma|F_o|$, where F_o and F_c are the observed and calculated structure factor amplitudes, respectively. R_{free}: The R factor calculated on a partial set that is not used in the refinement of the structure. Ramachandran plot parameters were obtained from Molprobity.

[a]The data used in Supplementary 9 in SI.

Supplementary Table 2: Selected bond angles and bond distances in the coordination structure of $Mn(CO)_3(wat)_2$ bounded by His15 in complete darkness structure.[a]

Bond distances (Å)		Bond angles (°)	
Mn-N^{ε}(His15)	2.26	C(CO_{ax})-Mn-N^{ε}(His15)	174.01
Mn-O(wat_{eq1})	2.15	C(CO_{eq1})-Mn-O(wat_{eq1})	173.51
Mn-O(wat_{eq2})	2.33	C(CO_{eq2})-Mn-O(wat_{eq4})	177.95
Mn-C(CO_{eq1})	1.94	Mn-C(CO_{eq1})-O(CO_{eq1})	178.05
Mn-C(CO_{eq2})	1.93	Mn-C(CO_{eq2})-O(CO_{eq2})	174.86
Mn-C(CO_{ax})	1.94	Mn-C(CO_{ax})-O(CO_{ax})	174.95
C-O(CO_{eq1})	1.19	wat_{eq1}-Mn-wat_{eq2}	85.69
C-O(CO_{eq2})	1.19	Wat_{eq2}-Mn-C(CO_{eq1})	88.17
C-O(CO_{ax})	1.19	C(CO_{eq2})-Mn-wat_{eq1}	94.51
O(wat_{eq1})-O^{ν} (Asp87)	2.74	C(CO_{eq1})-Mn-C(CO_{eq2})	91.55
O(wat_{eq1})-O^{δ} (Thr89)	3.14	Wat_{eq1}-Mn- N^{ε}(His15)	78.45
O(wat_{eq2})-N^{ε} (Arg14)	3.90	Wat_{eq2}-Mn- N^{ε}(His15)	82.76

[a]The data corresponds to the structure shown in Figure 3b. The bond distances and angles are measured using pymol.

Supplementary Table 3: Occupancy and B-factors (Å^2) of selected atoms in HEWL-$Mn(CO)_3(wat)_2$ at various conditions, with structures refined using a single model structure.

	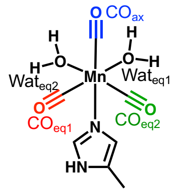	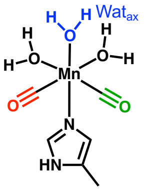	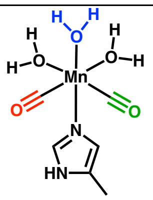	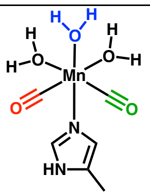	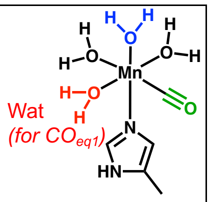
	**Darkness**	**10 ns20 µJ**	**100 ns20 µJ**	**1 µs20 µJ**	**1 µs40 µJ**
	**B-fact. (Occu.)**[a]	**B-fact. (Occu.)**[a]	**B-fact. (Occu.)**[a]	**B-fact. (Occu.)**[a]	**B-fact. (Occu.)**[a]
**N$^{\varepsilon 2}$ (His15)**	30.88 (1.0)	31.12 (1.0)	41.60 (1.0)	42.88 (1.0)	45.60 (1.0)
**Mn**	30.25 (1.0)	31.53 (0.9)	42.04 (0.9)	41.77 (0.85)	44.14 (0.65)
**CO$_{eq1}$, C**	30.56 (1.0)	29.98 (0.9)	40.58 (0.9)	43.21 (0.85)	45.53 (0.65)
**CO$_{eq1}$, O**	36.50 (1.0)	36.60 (0.9)	47.00 (0.9)	49.11 (0.85)	**(Wat)**
**CO$_{eq2}$, C**	30.20 (1.0)	31.70 (0.9)	38.83 (0.9)	40.81 (0.85)	40.91 (0.65)
**CO$_{eq2}$, O**	34.16 (1.0)	34.23 (0.9)	41.41 (0.9)	41.86 (0.85)	44.45 (0.65)
**CO$_{ax}$, C**	35.10 (1.0)	35.12 (0.9)	44.42 (0.9)	44.01 (0.85)	46.85 (0.65)
**CO$_{ax}$, O**	41.42 (1.0)	**(Wat$_{ax}$)**	**(Wat$_{ax}$)**	**(Wat$_{ax}$)**	**(Wat$_{ax}$)**
**Wat$_{eq1}$**	31.44 (1.0)	33.83 (0.9)	46.38 (0.9)	45.12 (0.85)	48.50 (0.65)
**Wat$_{eq2}$**	38.84 (1.0)	39.51 (0.9)	51.16 (0.9)	50.51 (0.85)	52.09 (0.65)
**Average B-factor**[b]					
**Main chain**	19.61	21.23	30.37	30.97	33.18
**Side chain**	23.76	25.19	35.42	35.88	37.10
**His15**	28.20	28.80	39.40	40.02	43.60
**Mn-unit**[c]	34.27	34.06	43.98	44.55	46.07

[a]B-factor (Å^2) and occupancy refinement: At the initial stage of model building, both B-factor and occupancy were allowed to refine freely in "Phenix refine". Finally, the occupancy of Mn ion was adjusted a little manually to have the B-factor close to the surrounding (N$^{\varepsilon 2}$ of His15). The occupancy of other coordinating ligands was kept same as Mn.

[b]The average B-factors of all protein side-chain, main-chain, His15 (side-chain) and Mn-unit were obtained from B-average in CCP4.

[c]Mn-unit represents the Mn ion with all coordinating ligands (wat or CO) except His15.

Supplementary Table 4. Measured changes in the difference density features during power titration.

	Δt = 10 ns			Δt = 1 µs			
	10 µJ	20 µJ	40 µJ	10 µJ	20 µJ	40 µJ	60 µJ
Scale e.Å^{-3}/σ	0.039	0.046	0.054	0.036	0.043	0.056	0.064
Mn	6.0 (0.235)	10.7 (0.495)	11.6 (0.626)	6.3 (0.237)	11.7 (0.497)	12.4 (0.692)	13.8 (0.884)
CO_{ax} σ (e.Å^{-3})	2.9 (0.114)	4.3 (0.199)	4.6 (0.246)	2.9 (0.109)	4.5 (0.191)	4.8 (0.268)	5.0 (0.327)
CO_{eq1} σ (e.Å^{-3})	0	1.9 (0.088)	2.5 (0.135)	0	2.4 (0.102)	4.0 (0.223)	3.6 (0.224)
CO_{eq2} σ (e.Å^{-3})	0	0	0	0	1.6 (0.066)	2.4 (0.134)	3.0 (0.186)

The values were obtained from the COOT visualization. For complete dark data, the difference density features are considered as zero. Δt is the delay time.

Supplementary Table 5. Comparison of bond distances (Å) around the Mn center between SFX and QM/MM results for species involved in the photoreaction.

	$MnCO_{ax}CO_{eq1}$ $CO_{eq2}Wat_2Im$ (1)	$MnWat_{ax}CO_{eq1}$ $CO_{eq2}Wat_2Im$ (2)	$MnWat_{ax}Wat_{eq1}$ $CO_{eq2}Wat_2Im$ (3)	Complete darkness **8WZF**	10ns 20μJ **8WZG**	1μs 40μJ **8WZV**
Mn-C@CO_{ax}	1.84			1.94		
Mn-O@Wat_{ax}		2.12	2.15		2.35	2.17
Mn-C@CO_{eq1}	1.81	1.78		1.94	1.94	
Mn-O@Wat_{eq1}			2.13			2.26
Mn-C@CO_{eq2}	1.79	1.77	1.76	1.93	1.94	1.95
Mn-O@Wat_{eq2}						
Mn-O@Wat_{wat1}	2.13	2.12	2.11	2.15	2.19	2.15
Mn-O@Wat_{wat2}	2.12	2.16	2.25	2.33	2.24	2.33
Mn-N@Im	2.12	2.07	2.07	2.26	2.22	2.45

Supplementary Table 6. Root-mean-square deviation (RMSD) of the Mn center among the QM/MM optimized structures and SFX structures. RMSD values are given in Angstrom(Å). The smallest values in comparisons with QM/MM and SFX results are underlined.

	$MnCO_{ax}CO_{eq1}$ $CO_{eq2}Wat_2Im$ **1**	$MnWat_{ax}CO_{eq1}$ $CO_{eq2}Wat_2Im$ **2**	$MnWat_{ax}Wat_{eq1}$ $CO_{eq2}Wat_2Im$ **3**	Complete darkness **8WZF**	10ns 20μJ **8WZG**	1μs 40μJ **8WZV**
**1**	0	0.108	0.157	0.209	0.281	0.398
**2**	0.108	0	0.146	0.240	0.250	0.394
**3**	0.157	0.146	0	0.237	0.257	0.374
**8WZF**	0.209	0.240	0.237	0	0.178	0.294
**8WZG**	0.281	0.250	0.257	0.178	0	0.218
**8WZV**	0.398	0.394	0.374	0.294	0.218	0

Supplementary Table 7. Comparison of the peak-top of absorption spectra in state **1** by using QM/MM methods with the experimental result. Different DFT functional, basis sets and QM region are adapted for the QM/MM methods.

Entry	(QM level for excited states: DFT functional/basis set, QM region)@ (QM level for the ground state calculation)	Peak-top / nm	Energy of the first excited state /eV (kcal)
1	B3LYP/DZVP, Mn center @B1	387	3.007 (69.3)
2	B3LYP/TZVP, Mn center @B1	384	3.025 (69.7)
3	B3LYP/TZVP+, Mn center @B1	386	3.009 (69.3)
4	CAM-B3LYP/DZVP, Mn center @B1	392	2.969 (68.4)
5	CAM-B3LYP/TZVP, Mn center @B1	388	2.998 (69.1)
6	CAM-B3LYP/TZVP+, Mn center @B1	389	2.983 (68.7)
7	CAM-B3LYP/DZVP, Mn center+Asp87 @B1 (QM: Mn center+Asp87)	397	2.963 (68.3)
8	CAM-B3LYP/TZVP, Mn center+Asp87 @B1(QM: Mn center+Asp87)	392	2.992 (68.9)
9	CAM-B3LYP/TZVP+, Mn center+Asp87 @B1(QM: Mn center+Asp87)	394	2.978 (68.6)
10	CAM-B3LYP/DZVP, Mn center+Asp87 @B2 (QM: Mn center+Asp87)	396	2.934 (67.6)
11	CAM-B3LYP/TZVP, Mn center+Asp87 @B2(QM: Mn center+Asp87)	392	2.967 (68.4)
12	CAM-B3LYP/TZVP+, Mn center+Asp87 @B2(QM: Mn center+Asp87)	394	2.952 (68.1)
10	Exp.	395	

Table S8. Energy differences (D*E* / kcal mol^{-1}) during the ligand-Water exchange reactions in HEWL-Mn(CO)$_n$Wat$_m$ calculated by using the NEB method. Each reaction pathway is optimized in the ground state (S$_0$).

Index	Reaction Step	D$E^{\neq}$(ex) [a)]	D$E^{\neq}$(g) [b)]	DE (g) [c)]
1	$\mathrm{Mn(CO)_3Wat_{eq1}\underline{Wat_{eq2}}Im}$ (**1**) + **Wat** →<br>$\mathrm{Mn(CO)_3Wat_{eq1}\mathbf{Wat}Im + \underline{Wat_{eq2}}}$	3.2	13.9	0
2	$\mathrm{Mn(CO)_2\underline{CO_{ax}}Wat_2Im}$ + **Wat** →<br>$\mathrm{Mn(CO)_2\mathbf{Wat}Wat_2Im}$ (**2**) + $\mathrm{\underline{CO_{ax}}}$	6.0	20.0	16.3
3	$\mathrm{Mn\underline{CO_{eq1}}(CO)_2Wat_2Im}$ + **Wat** →<br>$\mathrm{Mn\mathbf{Wat}(CO)_2Wat_2Im + \underline{CO_{eq1}}}$	2.2 (13.3)	21.9	18.9
4	$\mathrm{Mn\underline{CO_{eq2}}(CO)_2Wat_2Im}$ + **Wat** →<br>$\mathrm{Mn\mathbf{Wat}(CO)_2Wat_2Im + \underline{CO_{eq2}}}$	26.1	30.3	1.7
5	$\mathrm{Mn(CO)_3Wat_2\underline{Im}}$ + **Wat** →<br>$\mathrm{Mn(CO)_3Wat_2\mathbf{Wat} + \underline{Im}}$	21.1	22.5	16.0
6	$\mathrm{Mn\underline{CO_{eq1}}CO_{eq2}Wat_{ax}Wat_2Im}$ (**3**) + **Wat** →<br>$\mathrm{Mn\mathbf{Wat}CO_{eq2}Wat_{ax}Wat_2Im + \underline{CO_{eq1}}}$	10.2	24.1	17.5
7	$\mathrm{MnCO_{eq1}\underline{CO_{eq2}}Wat_{ax}Wat_2Im}$ + **Wat** →<br>$\mathrm{MnCO_{eq1}\mathbf{Wat}Wat_{ax}Wat_2Im + \underline{CO_{eq2}}}$	17.6	27.6	0.5
8	$\mathrm{Mn(CO)_2Wat_{ax}Wat_2\underline{Im}}$ + **Wat** →<br>$\mathrm{Mn(CO)_2Wat_{ax}Wat_2\mathbf{Wat}}$ (**5**) + $\mathrm{\underline{Im}}$	-2.9 (14.7)	26.9	22.0
9	$\mathrm{MnWat_{eq1}CO_{eq2}\underline{CO_{ax}}Wat_2Im}$ + **Wat** →<br>$\mathrm{MnWat_{eq1}CO_{eq2}\mathbf{Wat}Wat_2Im + \underline{CO_{ax}}}$	4.7 (7.0)	21.1	19.6
10	$\mathrm{MnWat_{eq1}\underline{CO_{eq2}}CO_{ax}Wat_2Im}$ + **Wat** →<br>$\mathrm{MnWat_{eq1}\mathbf{Wat}\ CO_{ax}Wat_2Im + \underline{CO_{eq2}}}$	13.8	32.0a	7.5
11	$\mathrm{MnWat_{eq1}CO_{eq2}CO_{ax}Wat_2\underline{Im}}$ + **Wat** →<br>$\mathrm{MnWat_{eq1}CO_{eq2}CO_{ax}Wat_2\mathbf{Wat} + \underline{Im}}$	21.1	26.3	17.2
12	$\mathrm{MnWat_2\underline{CO_{ax}}Wat_2Im}$ + **Wat** →<br>$\mathrm{MnWat_2\mathbf{Wat}Wat_2Im + \underline{CO_{ax}}}$	36.8	41.9	39.4
13	$\mathrm{MnWat_2CO_{ax}Wat_2\underline{Im}}$ + **Wat** →<br>$\mathrm{MnWat_2CO_{ax}Wat_2\mathbf{Wat} + \underline{Im}}$	21.1	40.8	31.1
14	$\mathrm{MnWat_{eq1}\underline{CO_{eq2}}Wat_{ax}Wat_2Im}$ + **Wat** →<br>$\mathrm{MnWat_{eq1}\mathbf{Wat}Wat_{ax}Wat_2Im + \underline{CO_{eq2}}}$	32.3	46.5	33.0
15	$\mathrm{MnWat_{eq1}Wat_{eq2}CO_{ax}Wat_2\underline{Im}}$ + **Wat** →<br>$\mathrm{MnWat_{eq1}Wat_{eq2}CO_{ax}Wat_2\mathbf{Wat}}$ (**4**) + $\mathrm{\underline{Im}}$	8.3	19.9	4.0
16	$\mathrm{MnWat_{eq1}Wat_{eq2}Wat_{ax}Wat_2\underline{Im}}$ + **Wat** →<br>$\mathrm{MnWat_{eq1}Wat_{eq2}Wat_{ax}Wat_2\mathbf{Wat} + \underline{Im}}$	50.6	42.7	33.4

[a)] Activation barrier in the first excited state along the ligand-water exchange reaction. Values in parenthesis are additional activation barrier in the ground state after the state transition from the first excited state to the ground state in the relaxation process.

[b)] Activation barrier in the ground state along the ligand-water exchange reaction.

[c)] Energy difference of the product state relative to the reactant state.

Supplementary Table 9: Atomic coordinates of the QM region in the intermediate states are provided in the XYZ format and in Å units. All the results are optimized at the B3LYP//DZVP level of theory in the QM/MM model used for the NEB calculations.

28				28			
Mn CO3 Wat3 Im (1)				del COeq1			
C	8.338996	-5.054998	10.908995	C	8.331996	-5.067998	10.917995
H	8.662996	-5.136998	9.869995	H	8.637996	-5.138998	9.873995
H	7.498996	-4.360998	10.963995	H	7.479996	-4.385998	10.980995
C	7.940996	-6.418997	11.388995	C	7.962996	-6.453997	11.382995
N	8.443996	-7.557996	10.782995	N	8.534996	-7.550996	10.755995
H	9.054996	-7.543996	9.964995	H	9.137996	-7.484996	9.937995
C	7.952996	-8.643996	11.421995	C	8.092996	-8.674996	11.350995
H	8.166996	-9.659995	11.149995	H	8.369996	-9.667995	11.043995
N	7.147997	-8.275996	12.413994	N	7.252997	-8.378996	12.338994
C	7.134997	-6.888997	12.400994	C	7.165997	-6.991997	12.369994
H	6.556997	-6.340997	13.123994	H	6.543997	-6.494997	13.096994
C	5.751997	-8.300996	14.912993	C	3.408998	-10.487995	15.497993
C	4.488998	-9.324996	12.918994	C	4.702998	-9.570995	12.852994
C	5.375997	-10.878995	14.805993	C	5.841997	-11.291995	14.502993
O	5.460997	-7.442996	15.625993	O	3.857998	-9.744995	16.238992
O	3.458998	-9.223996	12.401994	O	3.651998	-9.349996	12.404994
O	4.930998	-11.696994	15.475993	O	5.613997	-12.319994	14.994993
Mn	6.095997	-9.597995	13.690994	Mn	6.328997	-9.844995	13.534994
O	8.058996	-10.154995	14.276993	O	8.271996	-9.812995	14.567993
H	8.737996	-9.474996	14.117993	H	9.054996	-9.447996	14.116993
H	8.267996	-10.549995	15.152993	H	8.596996	-10.466995	15.233993
O	6.403997	-11.104995	12.237994	O	6.766997	-11.219995	11.995994
H	5.788997	-11.221995	11.451995	H	6.006997	-11.242995	11.351995
H	7.209997	-11.635995	12.129994	H	6.913997	-12.141994	12.265994
O	6.563997	-9.793995	17.391992	O	6.084997	-8.355996	14.988993
H	6.761997	-9.090996	18.062991	H	5.384997	-8.531996	15.635993
H	7.293997	-10.429995	17.502992	H	6.931997	-8.479996	15.463993
C	9.506996	-4.467998	11.738994	C	9.505996	-4.469998	11.739994
28				28			
del COax (2)				del COeq2			
C	8.344996	-5.032998	10.894995	C	8.339996	-5.048998	10.900995
H	8.678996	-5.121998	9.860995	H	8.646996	-5.094998	9.852995
H	7.505996	-4.331998	10.936995	H	7.472996	-4.381998	10.983995
C	7.935996	-6.402997	11.351995	C	8.021996	-6.449997	11.330995
N	8.451996	-7.519996	10.717995	N	8.665996	-7.507996	10.711995
H	9.057996	-7.470996	9.894995	H	9.251996	-7.415997	9.879995
C	8.000996	-8.622996	11.352995	C	8.315996	-8.651996	11.325995
H	8.257996	-9.624995	11.066995	H	8.712996	-9.609995	11.038995
N	7.207997	-8.282996	12.362994	N	7.452996	-8.411996	12.306994
C	7.158997	-6.898997	12.367994	C	7.264997	-7.034997	12.319994
H	6.580997	-6.368997	13.103994	H	6.613997	-6.567997	13.041994
C	5.911997	-8.312996	14.906993	C	5.901997	-8.572996	14.607993
C	4.698998	-9.322996	13.055994	C	3.090999	-12.242994	13.283994
C	3.678998	-12.263994	13.699994	C	5.792997	-11.152995	14.551993
O	5.488997	-7.517996	15.635993	O	5.482997	-7.701996	15.245993
O	3.650998	-9.129996	12.582994	O	2.477999	-11.284995	13.377994
O	2.714999	-12.107994	13.115994	O	5.308997	-11.972994	15.216993
Mn	6.331997	-9.574995	13.705994	Mn	6.525997	-9.853995	13.505994
O	8.392996	-10.068995	14.208993	O	8.485996	-10.165995	14.288993
H	9.047996	-9.361996	14.053993	H	9.099996	-9.405996	14.234993
H	8.674996	-10.507995	15.045993	H	8.613996	-10.559995	15.185993
O	6.472997	-11.254995	12.346994	O	7.231997	-11.327995	12.126994

H 5.861997 -11.256995 11.558995
H 7.354997 -11.521995 12.038994
O 5.923997 -11.097995 15.131993
H 6.632997 -11.285995 15.771993
H 5.727997 -11.926994 14.658993
C 9.510996 -4.463998 11.739994

28
del Imi
C 8.364996 -5.100998 10.917995
H 8.658996 -5.132998 9.866995
H 7.463996 -4.486998 11.018995
C 8.163996 -6.503997 11.409995
N 8.755996 -7.563996 10.745995
H 9.206996 -7.485996 9.828995
C 8.695996 -8.655996 11.535995
H 9.084996 -9.626995 11.267995
N 8.092996 -8.355996 12.676994
C 7.740996 -7.025997 12.608994
H 7.195997 -6.535997 13.404994
C 5.068998 -8.675996 14.776993
C 5.387997 -9.227996 12.282994
C 3.584998 -10.612995 13.505994
O 4.983998 -7.663996 15.324993
O 5.378997 -8.643996 11.289995
O 2.543999 -11.007995 13.213994
Mn 5.334997 -10.203995 13.816993
O 5.318997 -11.371995 15.577993
H 5.838997 -12.184994 15.491993
H 5.752997 -10.830995 16.288992
O 5.836997 -11.912994 12.694994
H 5.503997 -11.769994 11.764994
H 6.807997 -11.833994 12.655994
O 7.393997 -10.330995 14.072993
H 7.856996 -10.491995 14.916993
H 7.792996 -9.477996 13.619994
C 9.505996 -4.463998 11.735994

29
delCOax delCOeq2
C 8.332996 -5.038998 10.895995
H 8.637996 -5.080998 9.848995
H 7.471996 -4.370998 10.983995
C 7.985996 -6.435997 11.318995
N 8.579996 -7.506996 10.678995
H 9.151996 -7.420997 9.839995
C 8.224996 -8.640996 11.319995
H 8.561996 -9.619995 11.034995
N 7.417997 -8.370996 12.336994
C 7.256997 -6.993997 12.339994
H 6.640997 -6.509997 13.077994
C 5.930997 -8.513996 14.686993
C 4.135998 -12.653994 13.310994
O 5.384997 -7.706996 15.341993
O 3.135999 -12.101994 13.301994
Mn 6.646997 -9.726995 13.626994
O 8.623996 -10.035995 14.379993
H 9.247996 -9.289996 14.277993

H 7.301997 -11.028995 11.191995
H 8.131996 -11.593995 12.402994
O 4.899998 -9.712995 12.141994
H 4.086998 -9.838995 12.652994
H 4.889998 -10.448995 11.435995
C 9.506996 -4.463998 11.738994

29
delCOax delCOeq1 (3)
C 8.335996 -5.054998 10.901995
H 8.663996 -5.138998 9.864995
H 7.491996 -4.357998 10.942995
C 7.931996 -6.425997 11.373995
N 8.497996 -7.550996 10.801995
H 9.117996 -7.522996 9.987995
C 8.040996 -8.645996 11.454995
H 8.310996 -9.652995 11.186995
N 7.192997 -8.299996 12.418994
C 7.121997 -6.916997 12.373994
H 6.487997 -6.377997 13.056994
C 5.643997 -10.894995 17.688992
C 4.507998 -9.243996 12.984994
O 5.347997 -10.541995 18.722991
O 3.402998 -9.048996 12.621994
Mn 6.089997 -9.641995 13.598994
O 8.063996 -9.981995 14.581993
H 8.820996 -9.535996 14.167993
H 8.433996 -10.593995 15.263993
O 6.361997 -11.258995 12.254994
H 5.694997 -11.257995 11.493995
H 7.226997 -11.453995 11.857994
O 5.341997 -11.301995 14.732993
H 5.701997 -12.041994 14.208993
H 5.705997 -11.365995 15.633993
O 5.903997 -8.628996 15.456993
H 5.530997 -7.740996 15.555993
H 6.796997 -8.616996 15.850993
C 9.506996 -4.467998 11.737994

29
delCOeq1 delImi
C 8.360996 -5.018998 10.867995
H 8.705996 -5.096998 9.838995
H 7.526996 -4.313998 10.907995
C 7.956996 -6.380997 11.331995
N 8.435996 -7.516996 10.697995
H 9.012996 -7.497996 9.851995
C 8.067996 -8.599996 11.413995
H 8.289996 -9.621995 11.159995
N 7.368997 -8.203996 12.468994
C 7.283997 -6.833997 12.434994
H 6.772997 -6.289997 13.207994
C 4.241998 -9.383996 13.412994
C 3.941998 -11.754994 13.816993
O 3.512998 -8.616996 12.924994
O 3.029999 -12.391994 13.476994
Mn 5.284998 -10.625995 14.145993
O 6.700997 -12.015994 14.915993
H 7.383997 -11.652995 15.514993

H	8.850996	-10.462995	15.239993
O	7.266997	-11.420995	12.403994
H	6.994997	-11.405995	11.470995
H	8.230996	-11.577995	12.406994
O	6.095997	-11.388995	14.936993
H	6.484997	-12.192994	14.551993
H	6.525997	-11.269995	15.802993
O	4.899998	-9.819995	12.335994
H	4.102998	-10.011995	12.848994
H	4.886998	-10.454995	11.560995
C	9.502996	-4.463998	11.735994

32

delCOax delImi (5)

C	8.349996	-5.028998	10.878995
H	8.693996	-5.105998	9.847995
H	7.510996	-4.326998	10.913995
C	7.927996	-6.399997	11.328995
N	8.481996	-7.533996	10.750995
H	9.080996	-7.510996	9.922995
C	7.946996	-8.623996	11.356995
H	8.194996	-9.637995	11.097995
N	7.088997	-8.257996	12.301994
C	7.068997	-6.874997	12.287994
H	6.451997	-6.327997	12.977994
C	5.799997	-8.122996	14.813993
C	4.496998	-9.261996	13.003994
O	5.486997	-7.271997	15.538993
O	3.437998	-9.197996	12.523994
Mn	6.130997	-9.465996	13.679994
O	8.105996	-10.090995	14.220993
H	8.872996	-9.496996	14.021993
H	8.335996	-10.517995	15.072993
O	6.276997	-11.203995	12.437994
H	5.721997	-11.240995	11.605995
H	7.171997	-11.508995	12.219994
O	5.550997	-10.862995	15.181993
H	5.929997	-11.734994	14.985993
H	5.893997	-10.595995	16.077992
O	10.438995	-8.725996	13.555994
H	10.829995	-9.545996	13.155994
H	10.222995	-8.179996	12.780994
O	6.568997	-10.056995	17.508992
H	6.703997	-9.208996	18.001992
H	7.402997	-10.551995	17.649992
C	9.507996	-4.465998	11.734994

30

delCOax delCOeq1 delImi(4)

C	8.345996	-5.055998	10.901995
H	8.658996	-5.112998	9.859995
H	7.487996	-4.381998	10.974995
C	8.001996	-6.442997	11.361995
N	8.598996	-7.537996	10.761995
H	9.169996	-7.482996	9.913995
C	8.271996	-8.646996	11.465995
H	8.618996	-9.636995	11.212995

H	6.373997	-12.820994	15.350993
O	6.248997	-11.039995	12.332994
H	5.641997	-11.153995	11.529995
H	6.747997	-11.869994	12.415994
O	4.848998	-10.068995	16.161992
H	5.536997	-9.361996	16.102992
H	3.985998	-9.620995	16.172992
O	6.768997	-9.299996	14.596993
H	7.609996	-9.678995	14.907993
H	7.058997	-8.804996	13.465994
C	9.514996	-4.458998	11.735994

30

delCOeq1 delCOeq2 delCOax

C	8.324996	-5.074998	10.904995
H	8.634996	-5.112998	9.855995
H	7.462996	-4.406998	10.983995
C	7.981996	-6.480997	11.328995
N	8.631996	-7.542996	10.719995
H	9.221996	-7.449996	9.893995
C	8.237996	-8.688996	11.319995
H	8.606996	-9.662995	11.032995
N	7.357997	-8.445996	12.286994
C	7.187997	-7.065997	12.294994
H	6.510997	-6.606997	12.998994
C	4.205998	-12.174994	13.925993
O	3.345998	-12.191994	13.157994
Mn	6.576997	-9.801995	13.612994
O	8.425996	-9.483996	14.773993
H	9.186996	-9.111996	14.288993
H	8.793996	-10.182995	15.362993
O	7.207997	-11.606995	12.549994
H	6.721997	-11.664995	11.710994
H	8.136996	-11.807994	12.332994
O	4.825998	-9.733995	12.230994
H	4.017998	-9.995995	12.693994
H	4.867998	-10.388995	11.464995
O	5.793997	-8.247996	14.916993
H	5.301998	-8.788996	15.563993
H	6.630997	-8.064996	15.385993
O	6.157997	-10.935995	15.497993
H	5.745997	-11.785994	15.266993
H	6.954997	-11.132995	16.020992
C	9.501996	-4.470998	11.738994

33

delCOeq1 delCOeq2 delImi

C	8.351996	-5.063998	10.901995
H	8.662996	-5.101998	9.856995
H	7.479996	-4.408998	10.985995
C	8.034996	-6.461997	11.341995
N	8.632996	-7.546996	10.726995
H	9.201996	-7.478996	9.880995
C	8.286996	-8.665996	11.389995
H	8.625996	-9.653995	11.118995

N 7.484996 -8.332996 12.486994
C 7.305997 -6.963997 12.425994
H 6.698997 -6.441997 13.147994
C 4.035998 -9.038996 13.488994
O 3.336998 -8.296996 12.894994
Mn 4.768998 -10.378995 14.315993
O 5.338997 -12.326994 15.113993
H 5.621997 -12.622994 14.225993
H 6.167997 -12.291994 15.619993
O 5.601997 -11.373995 12.645994
H 5.210998 -11.333995 11.707994
H 6.458997 -10.916995 12.563994
O 2.854999 -11.362995 14.356993
H 2.909999 -11.773994 15.240993
H 2.177999 -10.660995 14.434993
O 4.395998 -9.767995 16.331992
H 5.298998 -9.692995 16.747992
H 4.070998 -8.852996 16.300992
O 6.745997 -9.877995 14.505993
H 7.034997 -9.594995 15.397993
H 7.077997 -9.210996 13.804993
C 9.506996 -4.464998 11.735994

31
Mn wat6
C 8.346996 -5.079998 10.906995
H 8.655996 -5.135998 9.861995
H 7.475996 -4.416998 10.975995
C 8.039996 -6.475997 11.380995
N 8.564996 -7.565996 10.707995
H 9.084996 -7.497996 9.832995
C 8.315996 -8.675996 11.440995
H 8.626996 -9.666995 11.149995
N 7.649996 -8.374996 12.544994
C 7.463996 -7.006997 12.513994
H 6.932997 -6.483997 13.295994
O 3.079999 -11.525995 14.099993
H 3.733998 -12.282994 14.243993
H 2.952999 -11.128995 14.982993
Mn 5.365997 -11.087995 14.088993
O 5.787997 -12.244994 15.888993
H 6.346997 -11.733994 16.505992
H 6.274997 -13.070994 15.719993
O 6.118997 -12.444994 12.599994
H 5.831997 -12.214994 11.691994
H 7.028997 -12.097994 12.673994
O 4.512998 -9.962995 12.426994
H 3.656998 -10.339995 12.724994
H 4.703998 -10.444995 11.572995
O 4.835998 -9.628995 15.555993
H 5.567997 -9.594995 16.231992
H 4.864998 -8.764996 15.118993
O 7.429997 -10.641995 13.973993
H 7.514996 -9.713995 13.574994
H 8.082996 -10.727995 14.689993
C 9.507996 -4.465998 11.735994

N 7.485996 -8.376996 12.411994
C 7.320997 -7.000997 12.384994
H 6.709997 -6.489997 13.109994
C 3.572998 -11.732994 13.699994
O 2.480999 -12.078994 13.431994
Mn 5.217998 -11.181995 13.966993
O 5.617997 -12.477994 15.619993
H 5.811997 -11.955994 16.417992
H 6.293997 -13.179994 15.568993
O 6.207997 -12.481994 12.611994
H 5.915997 -12.367994 11.682994
H 7.110997 -12.115994 12.656994
O 5.024998 -9.822995 12.320994
H 4.909998 -10.402995 11.514995
H 5.859997 -9.320996 12.186994
O 4.837998 -9.830995 15.547993
H 5.640997 -9.493996 16.016992
H 4.252998 -9.081996 15.370993
O 7.088997 -9.334996 17.025992
H 7.723996 -10.063995 17.182992
H 7.030997 -8.844996 17.889992
O 7.320997 -10.574995 14.073993
H 7.907996 -10.628995 14.845993
H 7.443996 -9.681995 13.659994
C 9.510996 -4.464998 11.738994

Supplementary Table 10: Atomic coordinates of the QM region in the intermediate states provided in the XYZ format and in Å units. All the results are optimized at the B3LYP//DZVP level of theory with the extended QM region including Asp87 in the QM/MM method used for the UV-vis absorption spectra.

```
35
Mn CO3 Wat3 Im Asp87 (1)
C     8.346   -5.063   10.903
H     8.668   -5.151    9.861
H     7.499   -4.370   10.941
C     7.951   -6.441   11.374
N     8.489   -7.564   10.765
H     9.102   -7.532    9.946
C     8.024   -8.664   11.395
H     8.267   -9.669   11.108
N     7.205   -8.327   12.386
C     7.155   -6.938   12.380
H     6.556   -6.399   13.097
C     3.031  -13.079    9.886
H     2.931  -13.655    8.962
H     2.736  -13.729   10.717
C     4.520  -12.693   10.090
O     4.805  -11.479   10.358
O     5.367  -13.622    9.987
C     5.799   -8.391   14.862
C     4.585   -9.471   12.878
C     5.495  -10.978   14.808
O     5.478   -7.520   15.548
O     3.556   -9.340   12.362
O     5.055  -11.792   15.482
Mn    6.193   -9.704   13.675
O     8.173  -10.168   14.293
H     8.824   -9.459   14.142
H     8.384  -10.561   15.169
O     6.614  -11.250   12.305
H     6.082  -11.366   11.461
H     7.515  -11.598   12.209
O     6.512   -9.841   17.444
H     6.732   -9.117   18.083
H     7.243  -10.479   17.545
H_L   9.169   -4.643   11.496
H_L   2.331  -12.227    9.856

36
Mn CO2 Wat4 Im Asp87 (2)
C     8.346   -5.041   10.884
H     8.683   -5.118    9.848
H     7.503   -4.347   10.929
C     7.945   -6.417   11.336
N     8.479   -7.547   10.736
H     9.099   -7.518    9.925
C     8.002   -8.639   11.375
H     8.241   -9.650   11.101
N     7.179   -8.285   12.357
C     7.139   -6.897   12.337
H     6.540   -6.351   13.046
C     3.051  -13.051    9.901
H     2.985  -13.635    8.977
H     2.754  -13.700   10.731
C     4.533  -12.641   10.145

34
delCOax delCOeq1 Asp87 (3)
C     8.326   -5.028   10.897
H     8.650   -5.099    9.858
H     7.485   -4.327   10.952
C     7.920   -6.410   11.342
N     8.494   -7.512   10.729
H     9.109   -7.461    9.914
C     8.009   -8.628   11.315
H     8.290   -9.624   11.028
N     7.142   -8.317   12.270
C     7.082   -6.935   12.297
H     6.453   -6.432   13.009
C     3.007  -13.101    9.865
H     2.896  -13.677    8.942
H     2.704  -13.758   10.688
C     4.514  -12.770   10.065
O     4.862  -11.573   10.300
O     5.305  -13.749    9.990
C     4.555   -9.456   12.781
O     3.486   -9.377   12.285
Mn    6.153   -9.650   13.480
O     8.216  -10.040   14.232
H     8.912   -9.382   14.040
H     8.549  -10.563   14.985
O     6.554  -11.417   12.392
H     6.025  -11.485   11.537
H     7.492  -11.525   12.174
O     5.595  -11.042   15.046
H     5.909  -11.903   14.723
H     6.107  -10.837   15.853
O     5.907   -8.185   15.052
H     4.960   -8.234   15.250
H     6.347   -8.571   15.853
H_L   9.157   -4.625   11.492
H_L   2.321  -12.237    9.841

37
delCOax delCOeq1 delImi Asp87 (4)
C     8.336   -5.054   10.907
H     8.647   -5.124    9.861
H     7.478   -4.377   10.969
C     7.990   -6.438   11.382
N     8.526   -7.547   10.752
H     9.066   -7.504    9.886
C     8.209   -8.646   11.475
H     8.518   -9.643   11.211
N     7.486   -8.313   12.535
C     7.338   -6.941   12.484
H     6.780   -6.405   13.235
C     2.986  -13.115    9.853
H     2.864  -13.686    8.929
H     2.686  -13.774   10.677
C     4.487  -12.779   10.030
O     4.816  -11.562   10.225
```

O 4.788 -11.456 10.537<br>O 5.410 -13.531 9.955<br>C 5.781 -8.168 14.792<br>C 4.572 -9.299 12.967<br>O 5.433 -7.305 15.495<br>O 3.496 -9.151 12.550<br>Mn 6.177 -9.516 13.699<br>O 8.184 -10.040 14.386<br>H 8.876 -9.382 14.197<br>H 8.441 -10.479 15.230<br>O 6.519 -11.239 12.564<br>H 5.982 -11.357 11.717<br>H 7.453 -11.431 12.375<br>O 5.509 -10.929 15.126<br>H 6.025 -11.743 15.025<br>H 5.645 -10.583 16.047<br>O 5.553 -9.587 17.524<br>H 5.027 -8.798 17.311<br>H 6.309 -9.212 18.028<br>H_L 9.166 -4.630 11.487<br>H_L 2.336 -12.215 9.856	O 5.293 -13.743 9.982<br>C 4.209 -9.119 13.443<br>O 3.631 -8.264 12.870<br>Mn 4.833 -10.487 14.312<br>O 5.328 -12.388 15.268<br>H 5.424 -12.831 14.398<br>H 6.238 -12.372 15.615<br>O 5.581 -11.661 12.727<br>H 5.286 -11.520 11.775<br>H 6.527 -11.443 12.730<br>O 2.871 -11.376 14.479<br>H 2.919 -11.613 15.424<br>H 2.229 -10.640 14.435<br>O 4.464 -9.768 16.299<br>H 5.367 -9.718 16.725<br>H 4.188 -8.840 16.228<br>O 6.836 -10.034 14.437<br>H 7.138 -9.718 15.309<br>H 7.093 -9.321 13.743<br>H_L 9.160 -4.630 11.493<br>H_L 2.313 -12.244 9.835

Supplementary References

1 Adams, P. D. *et al.* PHENIX: a comprehensive Python-based system for macromolecular structure solution. *Acta Crystallographica Section D* **66**, 213-221 (2010). https://doi.org:doi:10.1107/S0907444909052925

2 McCoy, A. J. *et al.* Phaser crystallographic software. *J. Appl. Crystallogr.* **40**, 658-674 (2007). https://doi.org:doi:10.1107/S0021889807021206

3 Vaney, M. C., Maignan, S., Ries-Kautt, M. & Ducruix, A. High-Resolution Structure (1.33 A) of a HEW Lysozyme Tetragonal Crystal Grown in the APCF Apparatus. Data and Structural Comparison with a Crystal Grown under Microgravity from SpaceHab-01 Mission. *Acta Crystallographica Section D* **52**, 505-517 (1996). https://doi.org:doi:10.1107/S090744499501674X

4 Razavet, M. *et al.* Tricarbonylmanganese(i)–lysozyme complex: a structurally characterized organometallic protein. *Chem. Commun.*, 2805-2807 (2007). https://doi.org:10.1039/B703887A

5 Nango, E. *et al.* A three-dimensional movie of structural changes in bacteriorhodopsin. *Science* **354**, 1552-1557 (2016). https://doi.org:10.1126/science.aah3497

6 Chen, V. B. *et al.* MolProbity: all-atom structure validation for macromolecular crystallography. *Acta Crystallographica Section D* **66**, 12-21 (2010). https://doi.org:doi:10.1107/S0907444909042073
